# Transient theory for scanning electrochemical microscopy of biological membrane transport: uncovering membrane–permeant interactions†

**DOI:** 10.1039/d4an00411f

**Published:** 2024-04-19

**Authors:** Siao-Han Huang, Shigeru Amemiya

**Affiliations:** a Department of Chemistry, University of Pittsburgh Pittsburgh PA 15260 USA amemiya@pitt.edu

## Abstract

Scanning electrochemical microscopy (SECM) has emerged as a powerful method to quantitatively investigate the transport of molecules and ions across various biological membranes as represented by living cells. Advantageously, SECM allows for the *in situ* and non-destructive imaging and measurement of high membrane permeability under simple steady-state conditions, thereby facilitating quantitative data analysis. The SECM method, however, has not provided any information about the interactions of a transported species, *i.e.*, a permeant, with a membrane through its components, *e.g.*, lipids, channels, and carriers. Herein, we propose theoretically that SECM enables the quantitative investigation of membrane–permeant interactions by employing transient conditions. Specifically, we model the membrane–permeant interactions based on a Langmuir-type isotherm to define the strength and kinetics of the interactions as well as the concentration of interaction sites. Finite element simulation predicts that each of the three parameters uniquely affects the chronoamperometric current response of an SECM tip to a permeant. Significantly, this prediction implies that all three parameters are determinable from an experimental chronoamperometric response of the SECM tip. Complimentarily, the steady-state current response of the SECM tip yields the overall membrane permeability based on the combination of the three parameters. Interestingly, our simulation also reveals the optimum strength of membrane–permeant interactions to maximize the transient flux of the permeant from the membrane to the tip.

## Abstract 2

The finte element simulation predicts that interactions of a biological membrane with a permeant can be determined quantitatively by employing transient scanning electrochemical microscopy to discriminate between one-step and two-step transport.

## Graphical abstract

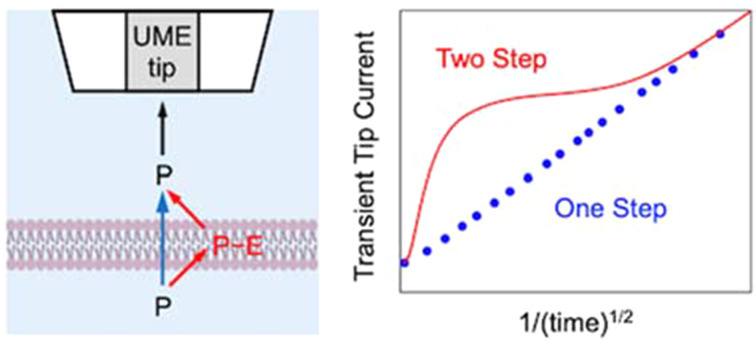

## 1 Introduction

A greater understanding of molecular and ion transport across biological membranes is imperative in many fields of biological^1^ and pharmaceutical^2^ sciences. Various physiological molecules and ions, as well as drug molecules, permeate through a biological membrane passively as mediated by simple transport,^3^ facilitated transport,^4^ or even both mechanisms.^5^ Simple transport is faster with a more hydrophobic permeant, which interacts more favorably with the hydrophobic core of a bilayer lipid membrane, also known as the Meyer–Overtone rule.^3^ By contrast, a small polar molecule or an ion must interact with carriers or channels in the membrane to go through facilitated transport.^4^ The carriers and channels are selective for physiological molecules and ions but are also targeted to the intracellular delivery of drug molecules.^6^ Facilitated transport has been quantitatively treated under steady states by the Michaelis–Menten model.^7^ The model is characterized by the maximum transport rate and the strength of interactions between a permeant and a carrier or a channel. Both parameters, however, are based on the combination of multiple thermodynamic and kinetic parameters, thereby limiting our quantitative understanding of the transport mechanism.

**Fig. 1:**
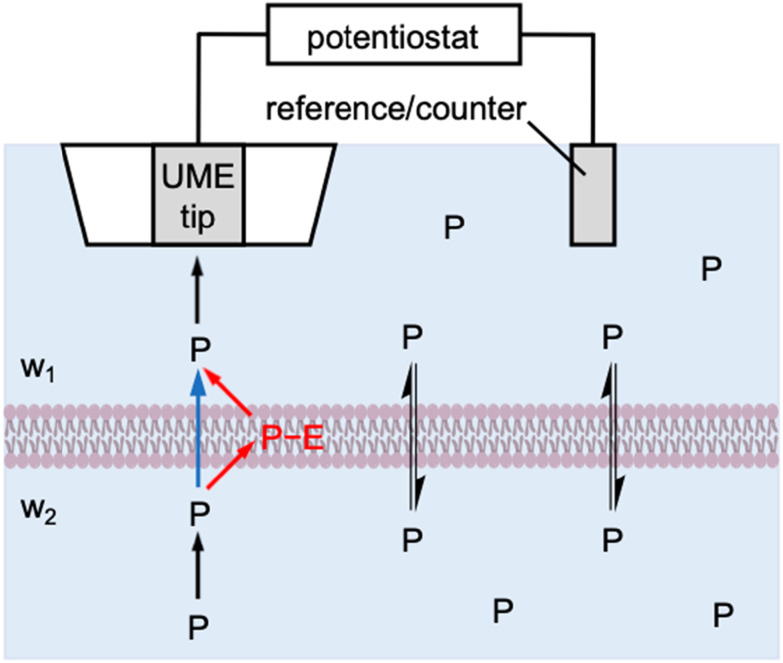
Scheme of the membrane transport of a permeant, *P*, as induced by an SECM tip through one-step (blue arrow) and two-step (red arrows) mechanisms.

Herein, we theoretically propose the transient operation mode of scanning electrochemical microscopy^8,9^ (SECM) to obtain quantitative insights into the interactions of molecular and ionic permeants with biological membranes. SECM has been successfully used to determine the permeability of cellular^10–12^ and neuronal^13,14^ membranes, the nuclear envelope through the nuclear pore complex,^15^ and the bacterial membrane through aquaporins.^16,17^ Experimentally, an ultramicroelectrode (UME) is positioned at the same side of the membrane as a reference/counter electrode to enable the *in situ* and non-contact measurement of membrane permeability (Fig. 1). In the induced mode of SECM,^18^ a permeant is initially added to the top solution (w_1_), transported across the membrane, and then pre-equilibrated with the bottom solution (w_2_). The permeant is amperometrically consumed by the tip approaching the membrane to induce and monitor the membrane flux of the permeant under high mass-transport conditions, thereby determining high membrane permeability. The resultant approach curves, however, are measured under steady states, which do not provide information about membrane–permeant interactions as confirmed in this work.

Specifically, we employ the finite element simulation to predict that SECM-based chronoamperometry^19^ enables the quantitative investigation of membrane–permeant interactions during the transport process. With this transient induced mode, an SECM tip is positioned at a fixed distance from the membrane to initiate the diffusion-limited reaction of a permeant by stepping the tip potential. The diffusion-limited tip current initially decays and then reaches a transient plateau owing to the permeant that is dissociated from the membrane and detected at the tip. Eventually, the tip current decays to the steady-state value that is controlled by the transport of the permeant through the membrane. Membrane–permeant interactions are modeled by a Langmuir isotherm to separately determine the strength and kinetics of the interactions as well as the concentration of interaction sites by SECM-based chronoamperometry. Interestingly, our simulation also manifests that membrane transport is fastest transiently when optimum membrane–permeant interactions result in half of the interaction sites in the membrane occupied by the permeant.

More broadly, this work is the first to reveal the power of transient SECM measurements for the investigation of membrane–permeant interactions. Previously, the chronoamperometric mode of SECM was employed to investigate the transport of small redox-active molecules across the nuclear envelope of an intact nucleus.^20^ This study, however, demonstrated that the small molecules freely diffuse through the nuclear pore complex as represented by the one-step mechanism (blue arrow in Fig. 1). Moreover, the adsorption of probe molecules on the solid/liquid interface^21^ and the Langmuir monolayer at the air/liquid interface^22,23^ were investigated quantitatively by employing SECM-based chronoamperometry. These studies, however, were limited to the one-step adsorption and surface diffusion of a probe molecule without membrane transport. By contrast, we will consider the two-step mechanism of membrane transport in this study (red arrows in Fig. 1) to quantify a membrane-bound permeant as an intermediate. Recently, the transient voltammetric mode of SECM detected an intermediate adsorbate to resolve multi-step electrodeposition on the electrode surface.^24^ The transient voltammetric approach was extended to discriminate between concerted and non-concerted mechanisms of adsorption-coupled electron-transfer reactions.^25^

## 2 Model

### 2.1 Membrane–permeant interactions

In this work, we consider membrane–permeant interactions under both steady-state and transient transport conditions. A Michaelis–Menten model has been employed for the kinetic description of facilitated transport under steady states.^2,5^ This model is modified in this work to result in a Langmuir isotherm for both simple and facilitated transport under steady-state and transient conditions (see ESI†). Specifically, membrane transport is defined by the two-step mechanism (Fig. 1) as

1 *P*(w_1_) + *E*(mem) ⇌ *P*−*E*(mem) ⇌ *P*(w_2_) + *E*(mem)

where *P* is the permeant, *E* is an interaction site in the membrane, *i.e.*, lipids, channels, carriers, *etc.*, and *P*−*E* is their complex. The rates of first and second steps, *v*_1_ and *v*_2_, are given by

2 *v*_1_ = *k*_ass_*c*_1,S_(*Γ*_S_ − *Γ*_PE_) − *k*_diss_*Γ*_PE_

3 *v*_2_ = *k*_diss_*Γ*_PE_ − *k*_ass_*c*_2,S_(*Γ*_S_ − *Γ*_PE_)

where *c*_1,S_ and *c*_2,S_ are the concentration of the permeant in w_1_ and w_2_ phases near the membrane, respectively, *k*_ass_ and *k*_diss_ are rate constants for association and dissociation of the permeant with the membrane, respectively, and *Γ*_S_ and *Γ*_PE_ are the membrane concentration of interaction site and its complex with the permeant, respectively. Eqn (2) and (3) are equivalent to the Langmuir isotherm under the equilibrium as given by

4 *βc*_i,S_ = *Γ*_PE_/(*Γ*_S_ − *Γ*_PE_)

where *i* = 1 or 2 and *β* is an association equilibrium constant as given by

5 *β* = *k*_ass_/*k*_diss_

By contrast, membrane–permeant interactions have not been considered in previous SECM studies of membrane transport based on the one-step mechanism (Fig. 1), *i.e.*,

6 *P*(w_1_) ⇌ *P*(w_2_)

The corresponding transport rate, *v*_mem_, is given by

7 *v*_mem_ = *k*_mem_(*c*_1,S_ − *c*_2,S_)

where *k*_mem_ is the transport rate constant.

### 2.2 Tip reaction

A tip reaction is driven at a diffusion-limited rate to induce the local flux of a permeant across the membrane under the tip. A redox-active is electrolyzed (*e.g.*, reduced to a reductant, *R*) at the conductive tip as given by^26,27^

8 *P*(w_1_) + *ne* ⇌ *R*(w_1_)

Alternatively, a permeant with an ionic charge of *z* may be present in the aqueous solution and detected by a micropipet tip filled with an organic electrolyte solution.^28,29^ With the ion-selective tip, the ionic permeant is transferred from the aqueous phase into the organic phase across the micropipet-supported liquid/liquid interface as given by

9 *P*^*z*^(w_1_) ⇌ *P*^*z*^(org)

The ion-transfer reaction is driven by controlling the interfacial potential. In either case, the permeant is amperometrically detected at the tip to yield a boundary condition as given by

10 *c*_1_ = 0

When the tip is far from the membrane, the tip current in the bulk solution, *i*_T,∞_, is given by

**Fig. 2:**
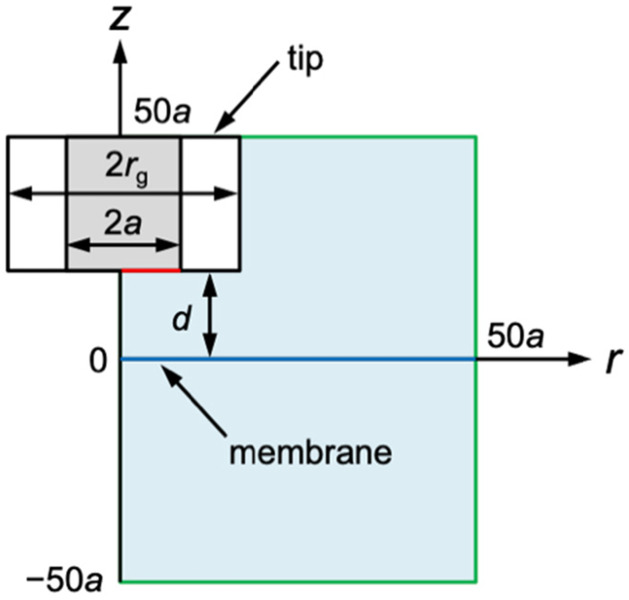
SECM diffusion problem of membrane transport in the cylindrical coordinate. The simulation space (light blue) is surrounded by eight boundaries (black, red, blue, and green lines). Boundary conditions at the tip and the membrane (red and blue lines, respectively) are given in the text. There is no normal flux at the symmetry axis and insulating surfaces (black lines). Simulation space limits are represented by green lines.

11 *i*_T,∞_ = 4*xmFDc*_0_*a*

where *x* is a function of *RG*^30^ (= *r*_g_/*a*; *a* and *r*_g_ are the inner and outer radii of a micropipet tip in Fig. 2), *m* = *n* or *z*, *F* is the Faraday constant, and *c*_0_ is the bulk concentration of the permeant.

### 2.3 SECM model

The diffusion-limited current at a disk-shaped SECM tip, *i*_T_, was simulated by solving an axisymmetric (2D) diffusion problem as defined in the cylindrical coordinate (Fig. 2). The origin of the axes was set at the membrane under the center of the tip. Initially, both solution phases, w_1_ and w_2_, contain a permeant at a bulk concentration of *c*_0_. The time-dependent diffusion of the permeant in each solution was defined by

12 ∂*c*_1_/∂*t* = *D*[∂^2^*c*_1_/∂*r*^2^ + (1/*r*)(∂*c*_1_/∂*r*) + ∂^2^*c*_1_/∂*z*^2^]

13 ∂*c*_2_/∂*t* = *D*[∂^2^*c*_2_/∂*r*^2^ + (1/*r*)(∂*c*_2_/∂*r*) + ∂^2^*c*_2_/∂*z*^2^]

where *c*_1_ and *c*_2_ are concentrations of the permeant at (*r*, *z*) in solutions 1 and 2, respectively, *D* is the diffusion coefficient of the permeant, *t* is time after the step of the tip potential in chronoamperometry, and ∂*c*_1_/∂*t* = ∂*c*_2_/∂*t* = 0 in steady-state approach curves.

Eqn (12) and (13) were solved by using the following initial and boundary conditions. Initial conditions at *t* = 0 are given for the permeant at any location in the bulk solutions as

14 *c*_1_(*t* = 0) = *c*_2_(*t* = 0) = *c*_0_

Accordingly, the entire membrane is equilibrated with the permeant initially to yield from eqn (4)

15 *Γ*_PE_(*t* = 0) = *βc*_0_*Γ*_S_/(1 + *βc*_0_)

A boundary condition for the permeant at the w_1_ side of the membrane was given by

16 *D*(∂*c*_1_/∂*z*) = *v*_1_ (or *v*_mem_)

For the opposite side of the membrane, the boundary condition was given by

17 *D*(∂*c*_2_/∂*z*) = *v*_2_ (or *v*_mem_)

In the two-step mechanism, the permeant is present in the membrane as a complex with an interaction site to yield the corresponding boundary condition as

18 ∂*Γ*_PE_/∂*t* = *v*_1_ + *v*_2_

Other boundary conditions for the permeant are given in Fig. 2 and ESI.†

We employed COMSOL Multiphysics (version 6.2, COMSOL, Burlington, MA) to solve the 2D SECM diffusion problem in dimensionless form. The normalized rate constant, *λ*_mem_, was defined for the one-step mechanism without membrane–permeant interactions as

19 *λ*_mem_ = *k*_mem_*a*/*D*

Three parameters for membrane–permeant interactions, *i.e.*, *k*_diss_, *β*, and *Γ*_S_, were normalized to yield

20 *λ* = *k*_diss_*a*^2^/*D*

21 *ρ* = *βc*_0_

22 *κ* = *Γ*_S_/*ac*_0_

where *λ* is the normalized dissociation constant, *ρ* is the normalized strength of membrane–permeant interactions, and *κ* is the normalized concentration of interaction sites in the membrane.

## 3 Results and discussion

### 3.1 Steady-state approach curves

We demonstrate both analytically and numerically that the multiple parameters of membrane–permeant interactions are combined to yield the steady-state transport rate of the permeant. Since *v*_1_ = *v*_2_ at steady states, the corresponding transport rate, *v*_ss_, is given for the two-step mechanism by using eqn (2) and (3) as

23 *v*_ss_ = *v*_1_ = *v*_2_ = (*v*_1_ + *v*_2_)/2 = *k*_ss_(*c*_1,S_ − *c*_2,S_)

with

24 *k*_ss_ = *k*_ass_*Γ*_S_/(2 + *βc*_0_)

where *k*_ss_ is the steady-state rate constant. Eqn (23) is equivalent to eqn (7) for the one-step mechanism without membrane–permeant interactions when *k*_ss_ = *k*_mem_. This equivalence indicates that membrane–permeant interactions are not relevant under steady states, where the corresponding rate constant is the combination of three parameters based on membrane–permeant interactions, *i.e.*, *k*_ass_, *Γ*_S_, *β*. Eqn (24) indicates that the measurement of *k*_ss_ at different *c*_0_ allows for the determination of *β* but not the resolution between *k*_ass_ and *Γ*_S_.

We employed the finite element simulation to confirm that the steady-state approach curves of SECM at the membrane are identical between one-step and two-step mechanisms. In this simulation, the tip current, *i*_T_, was normalized against the tip current in the bulk solution, *i*_T,∞_ (eqn (11)) and plotted against the normalized tip–substrate distance, *L*, *i.e.*, *d*/*a*. Identical steady-state approach curves were simulated for one-step and two-step mechanisms under steady states when *k*_mem_ = *k*_ss_ with eqn (24). This condition was defined in the normalized form in our simulation by combining eqn (24) with eqn (19)–(22) to yield

**Fig. 3:**
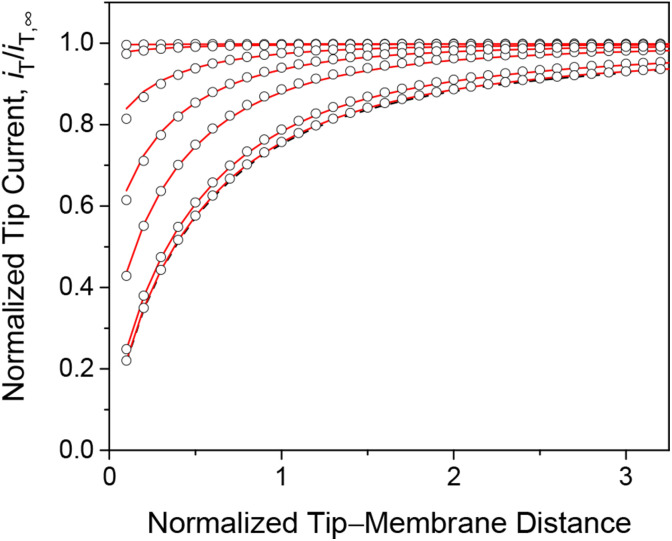
Steady-state approach curves simulated for two-step (solid lines) and one-step (circles) transport with *κ* = 10 and *ρ* = 1, and *λ* (= 0.3*λ*_mem_ in eqn (25)) = 10^2^, 1, 3 × 10^−1^, 1 × 10^−1^, 1 × 10^−2^, and 1 × 10^−3^ from the top to the bottom. Black dashed line represents an impermeable membrane.

25 *λ*_mem_ = *λκρ*/(2 + *ρ*)

where *λ* and *κ* are not separable. For instance, eqn (25) with *ρ* = 1 and *κ* = 10 corresponds to *λ* = 0.3*λ*_mem_ to obtain identical approach curves for both mechanisms with various *λ* values (Fig. 3). This result indicates that steady states do not allow for discrimination between one-step and two-step mechanisms or separate determination of membrane–permeant interaction parameters as combined in eqn (24).

### 3.2 Chronoamperometry of membrane–permeant interactions

**Fig. 4:**
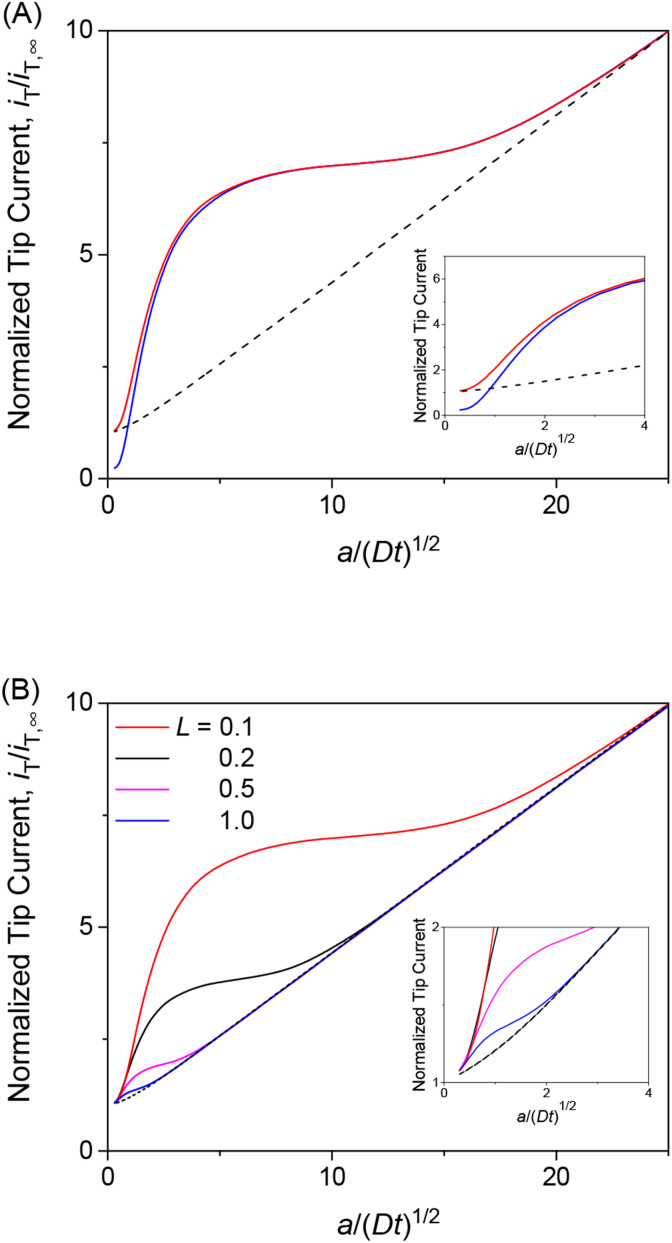
(A) Diffusion-limited chronoamperometric responses of two-step (red line) and one-step transport (black dashed line) as obtained with *κ* = 10, *ρ* = 1, *λ* (= 0.3*λ*_mem_ in eqn (25)) = 10^2^, and *L* = *d*/*a* = 0.1. Blue solid line employed the identical condition as two-step transport except *v*_2_ = 0. (B) Diffusion-limited chronoamperometric responses of two-step (solid lines) and one-step (dashed lines) transport with the same parameters as part (A) at *L* = 0.1, 0.2, 0.5, and 1.0. Responses of one-step transport are independent of *L*.

We employed the finite element method to find that a chronoamperometric tip response to a permeant is highly sensitive to membrane–permeant interactions in comparison with the steady-state counterpart. This finding was made by the finite element simulation of the tip current with various values of normalized parameters, *i.e.*, *λ*, *ρ*, and *κ*, as discussed in this section as well as in the sections below. For instance, we considered *κ* = 10 and *ρ* = 1 as well as *λ* = 10^2^, which represent fast association/dissociation kinetics to maintain the equilibrium Langmuir isotherm (eqn (4)). The resultant chronoamperogram was plotted against *a*/(*Dt*)^1/2^ to emphasize different tip currents between two-step and one-step mechanisms at long times (red solid and black dashed lines, respectively, in Fig. 4A). The higher current with the two-step mechanism is attributed to the dissociation of the membrane-bound permeant, which is transported through the tip–membrane gap and detected at the tip. The tip current decayed toward the steady-state value, which is identical with and without membrane–permeant interactions. At the late stage of *a*/(*Dt*)^1/2^ < 2, the tip current was enhanced also by the permeant transported from the bottom solution to the tip through the membrane. A lower tip current is expected without membrane transport, where the permeant in the bottom solution (w_2_) does not transfer into the membrane (blue solid line), *i.e.*, *v*_2_ = 0.

The tip current is enhanced by the permeant dissociated from the membrane more at a shorter tip–membrane distance with the two-step mechanism (solid lines in Fig. 4B). By contrast, the tip current is independent of the distance with the one-step mechanism (dashed lines). It should be noted that the initial concentration of the permeant in the membrane is equilibrated as given by eqn (4) and is independent of the tip–membrane distance.

### 3.3 Optimum interaction strength

**Fig. 5:**
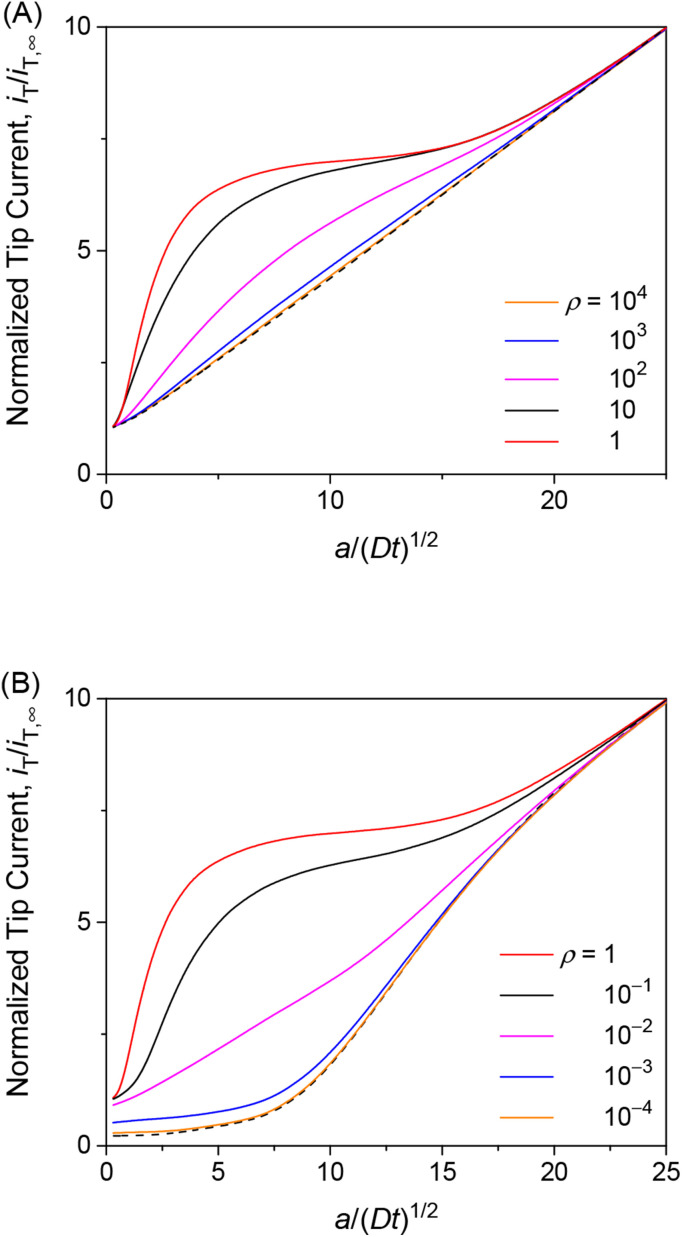
Diffusion-limited chronoamperometric responses of two-step transport with *λ* = 10^2^, *κ* = 10, and various *ρ* values (solid lines) at *L* = *d*/*a* = 0.1. Black dashed lines represent (A) diffusion-limited one-step transport with *λ*_mem_ = 3.3 × 10^3^ in eqn (25) with *λ* = 10^2^, *κ* = 10, and *ρ* = 1, and (B) an impermeable membrane.

The transient tip current is maximized when the strength of membrane–permeant interactions satisfies *ρ* (= *βc*_0_) = 1. We made this finding when the interaction sites are abundant (*κ* = 10) and the association/dissociation kinetics is fast enough (*λ* = 10^2^) to follow the Langmuir isotherm (eqn (4)). As *ρ* increases from 1, the initial concentration of the permeant bound to the membrane increases from *θ* (= *Γ*_PE_/*Γ*_S_) = 0.5. The tip current, however, was enhanced less with larger *ρ* (Fig. 5A). The tip current was barely enhanced with *ρ* = 10^3^, where all interaction sites were bound to the permeant. This result indicates that membrane–permeant interactions are too strong to dissociate the permeant from the membrane when the tip reaction significantly depletes the unbound permeant in the solution near the membrane. The tip current was lowered with *ρ* < 1 not only transiently (Fig. 5B) but also under steady states as demonstrated with approach curves (Fig. 3). The low *ρ* values prevent the transfer of the permeant into the membrane, thereby slowing down the overall membrane transport of the permeant. The resultant tip current is as low as expected at an impermeable membrane (black dashed line). It should be noted that chronoamperograms with *ρ* > 1 and *ρ* < 1 resemble each other (*i.e.*, *ρ* = 10 and 10^−1^ in Fig. 5A and B, respectively) but do not overlap with each other, thereby enabling the determination of unique *ρ*.

The finding of the optimum *ρ* value of 1 for the maximum tip current response (Fig. 5) is explainable as follows. When the kinetics of membrane–permeant interactions are fast enough to maintain the local equilibrium, a Langmuir isotherm (eqn (4)) is satisfied and differentiated to yield

26 ∂*Γ*_PE_/∂ ln *c*_1,S_ = *Γ*_PE_(*Γ*_S_ − *Γ*_PE_)

In the induced mode of SECM, the tip reaction of the permeant lowers *c*_1,S_ (Fig. 1), which is balanced by the dissociation of the permeant from the membrane to lower *Γ*_PE_ as expected from the Langmuir isotherm. The amount of the dissociated permeant per the lowered permeant concentration near the membrane is represented by ∂*Γ*_PE_/∂ ln *c*_1,S_ in eqn (26). The dissociated permeant is detected at the tip and is maximized when *Γ*_PE_ = *Γ*_S_/2, which is equivalent to *ρ* = *βc*_0_ = 1 initially in eqn (4). This condition corresponds to the standard state of surface adsorption based on a Langmuir isotherm.^31^ More qualitatively, this scenario of optimum interaction strength is analogous to that of buffer capacity.^32^ The concentration of the permeant near the membrane, *c*_1,S_, is buffered by permeant-free and permeant-bound interaction sites in the membrane. The buffer capacity is maximized when half of the interaction sites are bound to the permeant.

### 3.4 Interaction kinetics and site concentration

We also investigated the effects of interaction kinetics or interaction site concentrations by varying *λ* or *κ*, respectively, while maintaining the optimum *ρ* value of 1. Importantly, the choronoamperometric tip response depends on *κ*, *λ*, and *ρ* differently. This result implies that each parameter can be determined separately from a chronoamperometric response. This task can be facilitated further by measuring steady-state rate constants at various concentrations of the permeant in the solution, which allows for the determination of *ρ* (see eqn (25)). Accordingly, the resolution only between *κ* and *λ* by chronoamperometry is required.

**Fig. 6:**
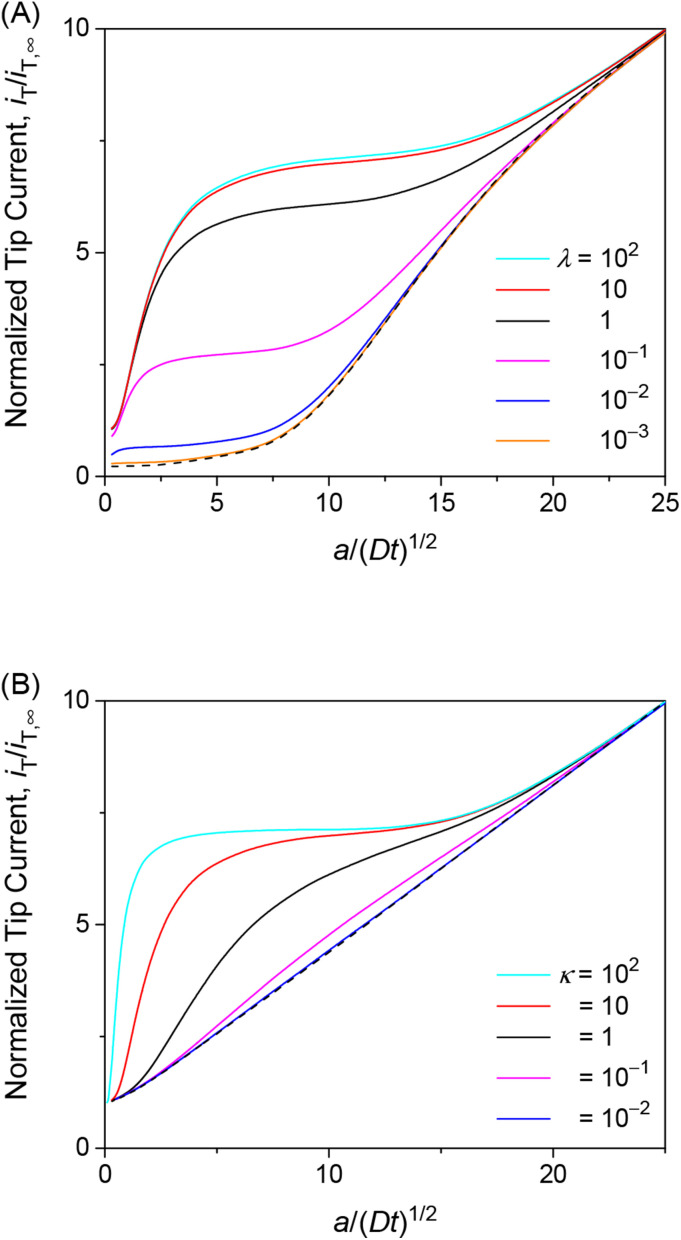
Diffusion-limited chronoamperometric responses of two-step transport with *ρ* = 1 as well as (A) *κ* = 10 and various *λ* or (B) *λ* = 10^2^ and various *κ* at *L* = *d*/*a* = 0.1. Black dashed lines represent (A) an impermeable membrane and (B) diffusion-limited one-step transport with *λ*_mem_ = 3.3 × 10^3^ in eqn (25) with *λ* = 10^2^, *κ* = 10, and *ρ* = 1.

The chronoamperometric response of the tip current to the permeant is lowered as *λ* or *κ* becomes lower. Lower *λ* kinetically limits the dissociation of the permeant from the membrane to lower the tip current (Fig. 6A). The tip current becomes as low as expected with an impermeable membrane without permeant–membrane interactions when *λ* < 0.1 prevents the dissociation of permeants from the membrane. The corresponding *λ* values also yield steady-state approach curves without an effect of membrane transport, *i.e.*, negative feedback approach curves (Fig. 3). The tip current is maximized and limited by the availability of membrane-bound permeants when *λ* > 10^2^. By contrast, lower *κ* corresponds to less permeant associated with the membrane, thereby lowering the tip current (Fig. 6B). Very small *κ*, however, still allows for the membrane transport of permeants to maintain the tip current as expected for a one-step mechanism without membrane–permeant interactions. The tip current increases monotonically with larger *κ*, where membrane-bound permeants are more abundant.

The unique dependence of *λ* and *κ* on the permeant concentration, *c*_0_, and the tip radius, *a*, will facilitate the determination of *λ*, *κ*, and *ρ* from chronoamperograms. Specifically, *λ* is independent of *c*_0_ in contrast to *κ* and *ρ*. Lower *c*_0_ increases *κ* to enhance the tip current response (Fig. 6B). Lower *c*_0_ decreases *ρ* to further enhance the tip current response when *ρ* > 1 (Fig. 5A) or counterbalance the enhanced tip current when *ρ* < 1 (Fig. 5A), thereby enabling us to assess whether *ρ* is larger or smaller than 1. By contrast, *ρ* is independent of *a* in contrast to *κ* and *λ*. A smaller tip decreases *λ* to lower the tip current, which can be counterbalanced by the enhancement of the tip current owing to higher *κ* with lower *a*. The chronoamperometric tip response, however, is lowered overall with a smaller tip because the square dependence of *λ* on *a* is stronger than the linear dependence of *κ* on *a*.

### 3.5 Experimental feasibility

The chronoamperometric mode of SECM proposed in this work is feasible experimentally as demonstrated by investigating the transport of small redox-active molecules across the nuclear envelope of an intact nucleus.^20^ The previous study yielded a good fit of an experimental chronoamperogram with a simulated one to ensure the lack of interactions between the nuclear envelope and a permeant. The permeant is too hydrophilic to interact with the nuclear envelope and is also small enough to freely diffuse through the nuclear pore complex.^26,27^ The high-quality chronoamperogram was obtained by using a 10 μm-diameter Pt tip and a relatively high concentration of a permeant (2.5 mM). Subsequently, the faradaic tip current was much higher than the non-faradaic tip current, thereby eliminating the need for background subtraction. Moreover, a lower diffusion coefficient of the permeant in the nucleus (w_2_) than in the outer aqueous solution (w_1_) was considered in the model and determined experimentally.^20^ The SECM model developed in this work can be extended for different diffusion coefficients of a permeant in both solution phases. Moreover, our model can be generalized by considering permeant–permeant interactions as a Frumkin-type isotherm^33^ and the surface diffusion of the permeant on the membrane. Such a generalized model was developed to quantitatively assess the time-dependent tip current response to the redox-active molecule that was involved in electron-transfer and adsorption reactions on the substrate electrode.^25^

It should be noted that the values of dimensionless parameters, *λ*, *κ*, *ρ*, used in this work are experimentally relevant. Recently, we determined *λ* = ∼20, *κ* = ∼15, and *ρ* = ∼1 for interactions between neurotoxic polydipeptides and nuclear pore complexes (NPCs) at the cell nucleus by SECM-based chronoamperometry.^34^ The association of the polydipeptides with the NPCs was observed qualitatively but was not assessed quantitatively by super-resolution fluorescence microscopy.^35^

## 4 Conclusions

This work is the first to predict that transient SECM measurements can uncover membrane–permeant interactions during the transport process. By contrast, the steady-state operation of SECM, normally, approach curve measurements, allows for the determination of only membrane permeability, which is based on the combination of multiple interaction parameters, *i.e.*, interaction strength, interaction kinetics, and the concentration of interaction sites in the Langmuir-type isotherm. We predict that these three parameters will be determinable separately by measuring the chronoamperometric response of the SECM tip positioned at a fixed short distance from the membrane. The proposed transient SECM method is experimentally feasible^20^ and will be applied to provide unprecedented insights into the thermodynamics and kinetics of biological membrane transport. A goal of such an application will be to test the prediction that the optimum membrane–permeant interactions with *ρ* = 1 maximize the transient membrane flux of a permeant. This prediction implies that permeant-interacting membrane components can serve as buffers to dynamically maintain the physiological concentration of a permeant, which is consumed or produced through various biological processes.

## Conflicts of interest

There are no conflicts to declare.

## Supplementary Material

AN-149-D4AN00411F-s001
