# Supplementary material for: Transient theory for scanning electrochemical microscopy of biological membrane transport: uncovering membrane–permeant interactions
Source: Analyst. 2024 Apr 19;149(11):3115–22. doi: 10.1039/d4an00411f (PMC11131039; doi:10.1039/d4an00411f)
Supplement: AN-149-D4AN00411F-s001 [file AN-149-D4AN00411F-s001.pdf]

Electronic Supplementary Material (ESI) for Analyst.
This journal is © The Royal Society of Chemistry 2024

Supplementary Information

Transient Theory for Scanning Electrochemical Microscopy of Biological Membrane Transport: Uncovering Permeant–Membrane Interactions

Siao-Han Huang, and Shigeru Amemiya*

Department of Chemistry, University of Pittsburgh, 219 Parkman Avenue, Pittsburgh, Pennsylvania, 15260, United States

* To whom correspondence should be addressed. E-mail: amemiya@pitt.edu. Fax: 412-624-8611.

Contents:

1. Transport model

2. Finite element simulation

Transport Model. We derived a Langmuir-type model for the reversible membrane transport of molecules and ions by modifying the Michaelis-Menten model of unidirectional membrane transport. With the Michaelis-Menten model, a permeant associated with a membrane component reversibly in the first step but is transported uni-directionally in the second step from the top phase (w_1 in Fig. 1) to the bottom phase (w_2) as given by

$$P(w_1) + E(mem) \rightleftharpoons PE(mem) \rightarrow E(mem) + P(w_2) \quad \text{(S-1)}$$

The respective rates are given by

$$v_{1,1\rightarrow2} = k_{on}c_{1,S}(\Gamma_S - \Gamma_{PE}) - k_{off}\Gamma_{PE} \quad \text{(S-2)}$$

$$v_{2,1\rightarrow2} = k_{cat}\Gamma_{PE} \quad \text{(S-3)}$$

where k_{on}, k_{off}, and k_{cat} are the corresponding rate constants. Since $v_{1,MM} = v_{2,MM}$ under steady states, the stead-state rate, $v_{ss,1\rightarrow2}$, is given by the familiar Michaelis–Menten expression as

$$v_{SS,1\rightarrow2} = \frac{V_{max}c_{1,S}}{c_{1,S} + K_M} \quad \text{(S-4)}$$

with

$$V_{max} = k_{cat}\Gamma_S \quad \text{(S-5)}$$

$$K_M = \frac{k_{off} + k_{cat}}{k_{on}} \quad \text{(S-6)}$$

Eqs S-5 and S-6 indicate that V_{max} and K_M are the convolutions of a few parameters, which can not be resolved under steady states. In addition, we also consider the reverse direction of membrane transport from the bottom phase (w_2 in Fig. 1) to the top phase (w_1) as

$$P(w_2) + E(mem) \rightleftharpoons P\text{–}E(mem) \rightarrow E(mem) + P(w_1) \quad \text{(S-7)}$$

Both directions are considered to modify eqs S-2 and S-3 as

$$v_{1,MM} = k_{on}c_{1,S}(\Gamma_S - \Gamma_{PE}) - k_{off}\Gamma_{PE} - k_{cat}\Gamma_{PE} \quad \text{(S-8)}$$

$$v_{2,MM} = k_{cat}\Gamma_{PE} - k_{on}c_{2,S}(\Gamma_S - \Gamma_{PE}) + k_{off}\Gamma_{PE} \tag{S-9}$$

With $k_{ass} = k_{on}$ and $k_{diss} = k_{off} + k_{cat}$, eqs S-8 and S-9 are equivalent to eqs 2 and 3 of the Langmuir-type model.

Finite Element Simulation. The current response of a disk-shaped tip in the SECM configuration was simulated by solving an axisymmetric (2D) diffusion problem as defined in a cylindrical coordinate (Fig. 2). We employed COMSOL Multiphysics (version 6.2, COMSOL, Inc., Burlington, MA) to solve the 2D SECM diffusion problem in dimensionless form. The details of the simulation are summarized in the report generated by COMSOL (see below). Study 1 simulates the steady-state tip current at various tip–membrane distances to yield an approach curve. Study 2 simulates the transient tipp current at various times to yield a chronoamperogram at a fixed tip–membrane distance.

The dimensionless equations are defined as follows. Eqs 11 and 12 for the diffusion of permeants were defined by dimensionless parameters as

$$\frac{\partial C_1}{\partial \tau} = \frac{\partial^2 C_1}{\partial R^2} + \frac{1}{R}\frac{\partial C_1}{\partial R} + \frac{\partial^2 C_1}{\partial Z^2} \tag{S-10}$$

$$\frac{\partial C_2}{\partial \tau} = \frac{\partial^2 C_2}{\partial R^2} + \frac{1}{R}\frac{\partial C_2}{\partial R} + \frac{\partial^2 C_2}{\partial Z^2} \tag{S-11}$$

where

$$C_1 = \frac{c_1}{c_0} \tag{S-12}$$

$$C_2 = \frac{c_2}{c_0} \tag{S-13}$$

$$\tau = \frac{Dt}{a^2} \tag{S-14}$$

$$R = \frac{r}{a} \tag{S-15}$$

$$Z = \frac{z}{a} \tag{S-16}$$

In addition, geometric parameters were defined by using dimensionless parameters as

$$L = \frac{d}{a} \tag{S-17}$$

This problem was solved numerically to calculate the normalized tip current, $i_T/i_{T,\infty}$, which was set to 1 at $L = 25$.

Boundary conditions for the permeant at the membrane (eqs 13–15) were also defined by using dimensionless parameters as

$$\frac{\partial C_1}{\partial Z} = \kappa\lambda\left[\rho C_1\left(1 - \theta_{PE}\right) - \theta_{PE}\right] \tag{S-18}$$

$$\frac{\partial C_2}{\partial Z} = \kappa\lambda\left[\rho C_2\left(1 - \theta_{PE}\right) - \theta_{PE}\right] \tag{S-19}$$

$$\frac{\partial \theta_{PE}}{\partial \tau} = \lambda\left[\rho\left(C_1 + C_2\right)\left(1 - \theta_{PE}\right) - 2\theta_{PE}\right] \tag{S-20}$$

where λ, ρ, and κ are given by eqs 17–19, respectively.

Report date

Apr 11, 2024, 12:51:57 PM

Contents

1. Global Definitions
1.1. Parameters
2. Component 1
2.1. Definitions
2.2. Geometry 1
2.3. Transport of Diluted Species
2.4. Transport of Diluted Species 2
2.5. General Form Boundary PDE
2.6. Mesh 1
3. Study 1
3.1. Parametric Sweep
3.2. Stationary
4. Study 2
4.1. Time Dependent
5. Results
5.1. Data Sets
5.2. Derived Values
5.3. Tables
5.4. Plot Groups

1. Global Definitions

Date	Apr 11, 2024, 11:01:01 AM

Global settings

Name	Transient SECM of Membrane Transport.mph
Path	E:\Transient SECM of Membrane Transport.mph
Version	COMSOL Multiphysics 6.2 (Build: 339)

Used products

COMSOL Multiphysics

Computer information

CPU	Intel64 Family 6 Model 158 Stepping 13, 8 cores, 15.79 GB RAM
Operating system	Windows 10

1.1. Parameters

Parameters 1

Name	Expression	Value	Description

lamda	0.1 [m/s]	0.1 m/s	normalized rate constant
rho	1	1	
kappa	10	10	
L	0.1 [m]	0.1 m	normalized tip-membrane distance
RG	1.4[m]	1.4 m	normalized tip outer radius
c0	1 [mol/m^3]	1 mol/m³	
Dg	0 [m^2/s]	0 m²/s	diffusion coefficient of permeant on the membrane

2. Component 1

Settings

Description	Value
Avoid inverted elements by curving interior domain elements	Off

2.1. Definitions

2.1.1. Variables

Variables 1

Selection

Geometric entity level	Entire model

Name	Expression	Unit	Description
V1	kappa*lamda*(rho*c1*(1 - u) - c0*u)	mol²/(m⁴·s)	
V2	kappa*lamda*(rho*c2*(1 - u) - c0*u)	mol²/(m⁴·s)	

2.1.2. Probes

Boundary Probe 1

Probe type	Boundary probe

Selection

Geometric entity level	Boundary
Selection	Geometry geom1: Dimension 1: Boundary 5

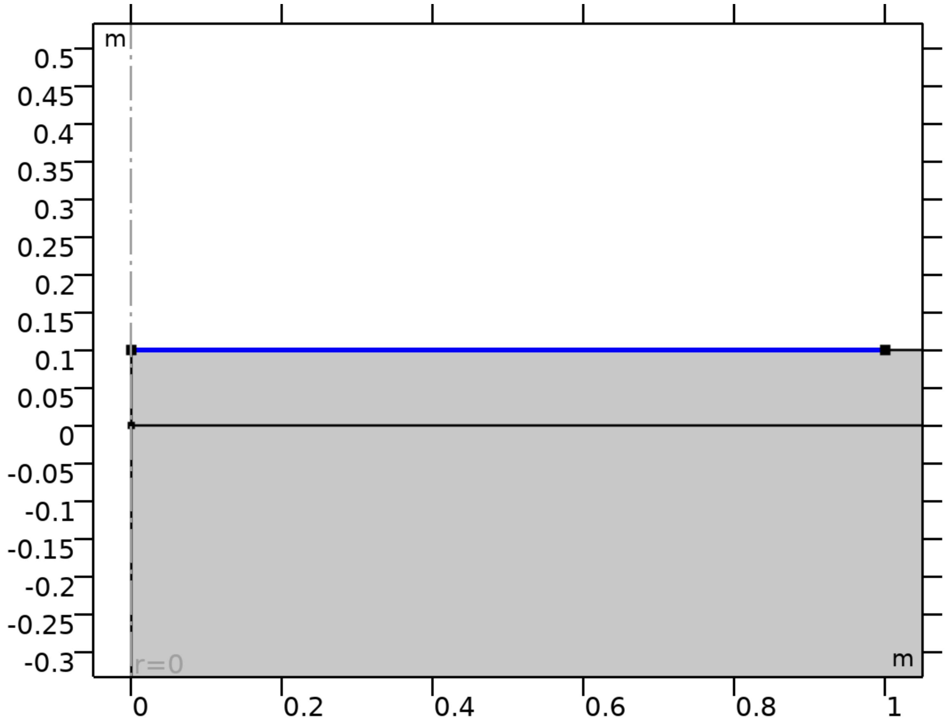

Selection

2.1.3. Nonlocal Couplings

Integration 1

Coupling type	Integration
Operator name	intop1

Selection

Geometric entity level	Boundary
Selection	Geometry geom1: Dimension 1: Boundary 5

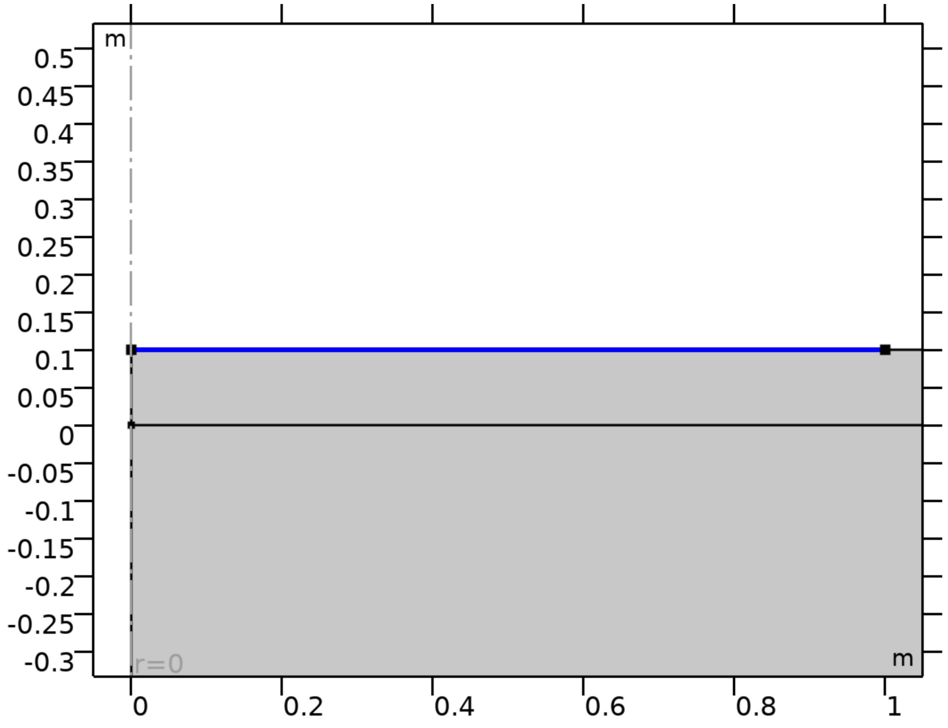

Selection

2.1.4. Coordinate Systems

Boundary System 1

Coordinate system type	Boundary system
Tag	sys1

Coordinate names

First	Second	Third
t1	to	n

2.2. Geometry 1

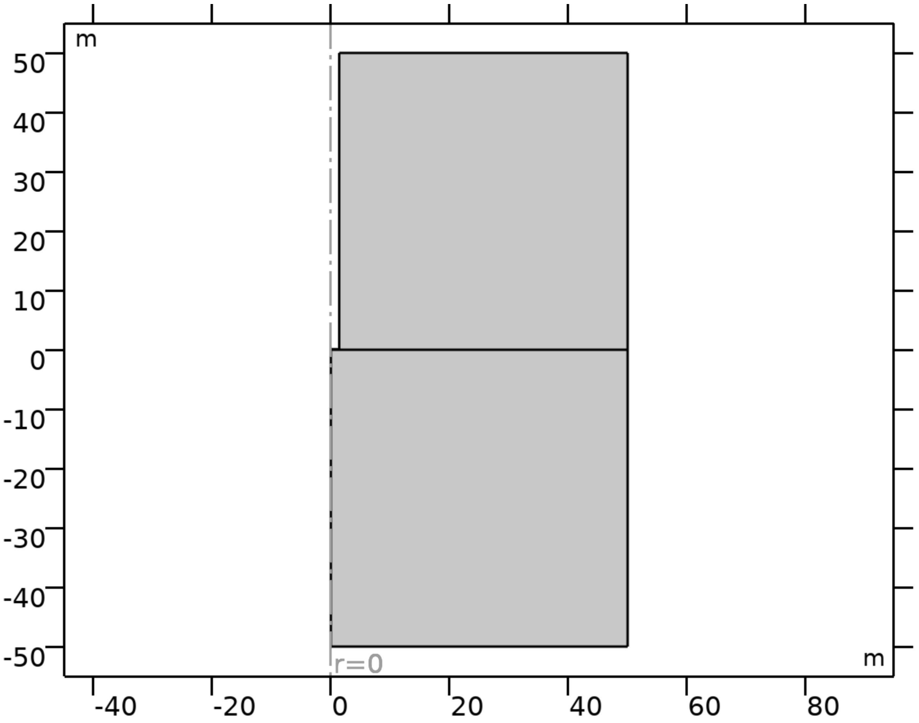

Geometry 1

Units

Length unit	m
Angular unit	deg

2.3. Transport of Diluted Species

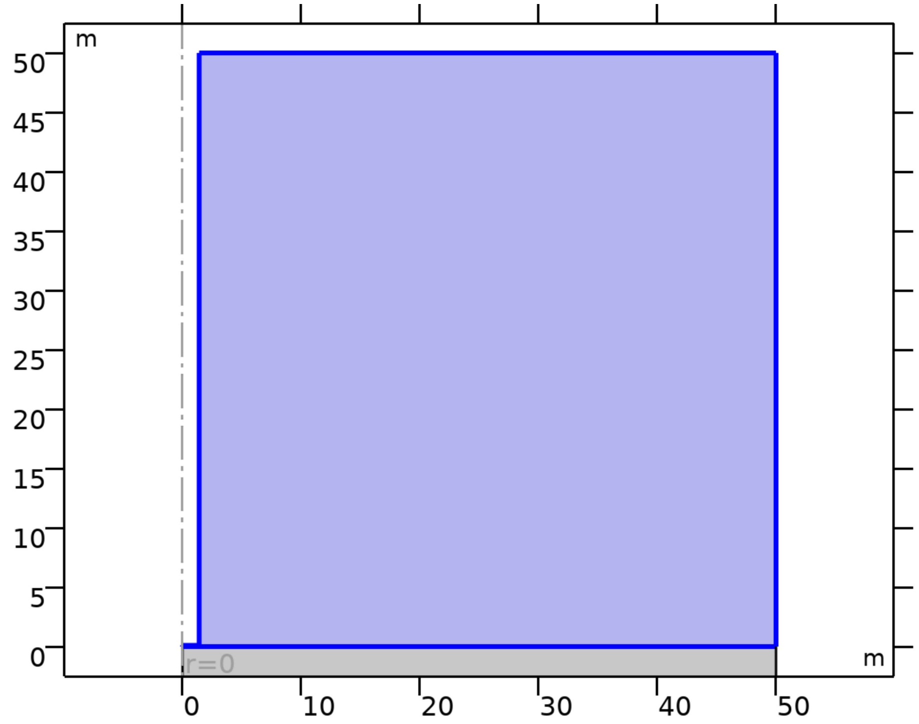

Transport of Diluted Species

Equations

$$\nabla \cdot \mathbf{J}_i + \mathbf{u} \cdot \nabla c_i = R_i$$

$$\mathbf{J}_i = -D_i \nabla c_i$$

Features

Transport Properties 1	Domain
Axial Symmetry 1	Boundary
No Flux 1	Boundary
Initial Values 1	Domain
Concentration 1	Boundary
Concentration 2	Boundary
Flux 1	Boundary

2.4. Transport of Diluted Species 2

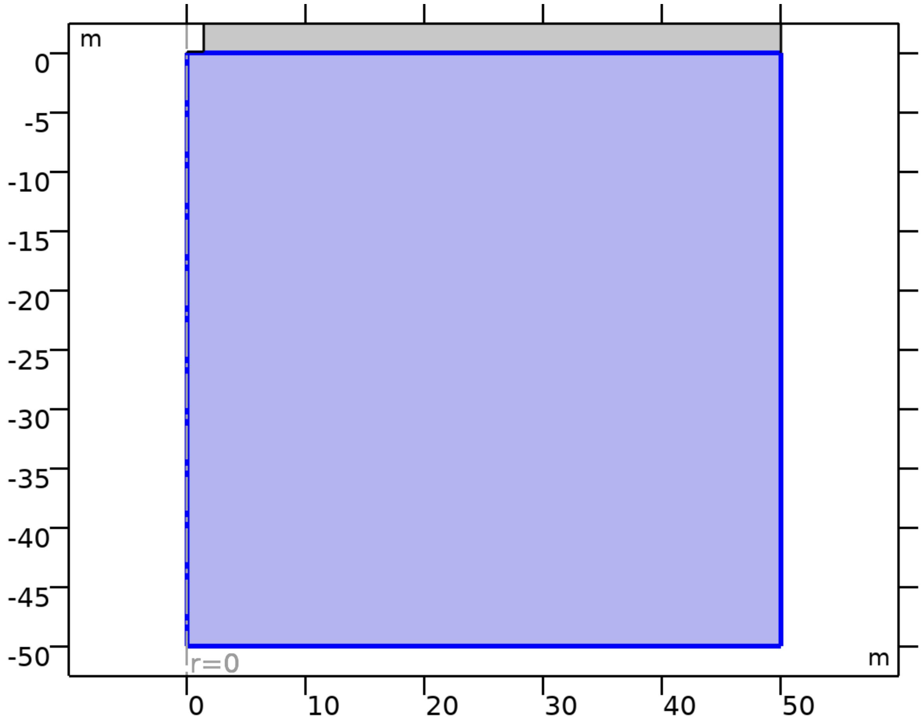

Transport of Diluted Species 2

Equations

$$\nabla \cdot \mathbf{J}_i + \mathbf{u} \cdot \nabla c_i = R_i$$

$$\mathbf{J}_i = -D_i \nabla c_i$$

Features

Transport Properties 1	Domain
Axial Symmetry 1	Boundary
No Flux 1	Boundary
Initial Values 1	Domain
Concentration 1	Boundary

Flux 1	Boundary

2.5. General Form Boundary PDE

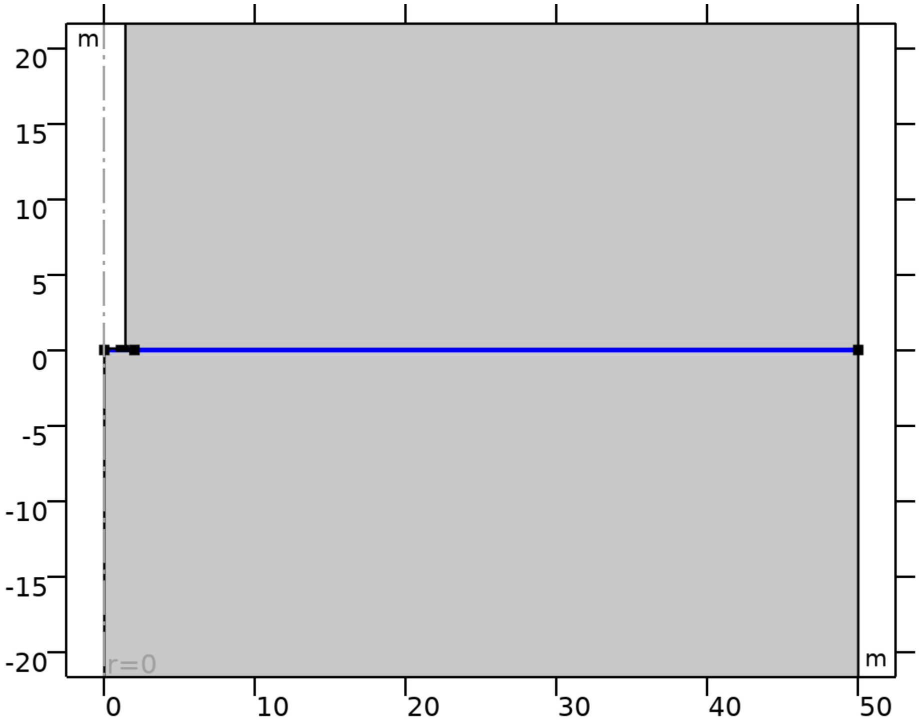

General Form Boundary PDE

Equations

$$\nabla_T \cdot \Gamma = f$$

$$\nabla_T = (\mathbf{I} - \mathbf{n}\mathbf{n}^T)[\frac{\partial}{\partial r}, \frac{\partial}{\partial z}]$$

Features

Name	Level
General Form PDE 1	Boundary
Initial Values 1	Boundary

2.6. Mesh 1

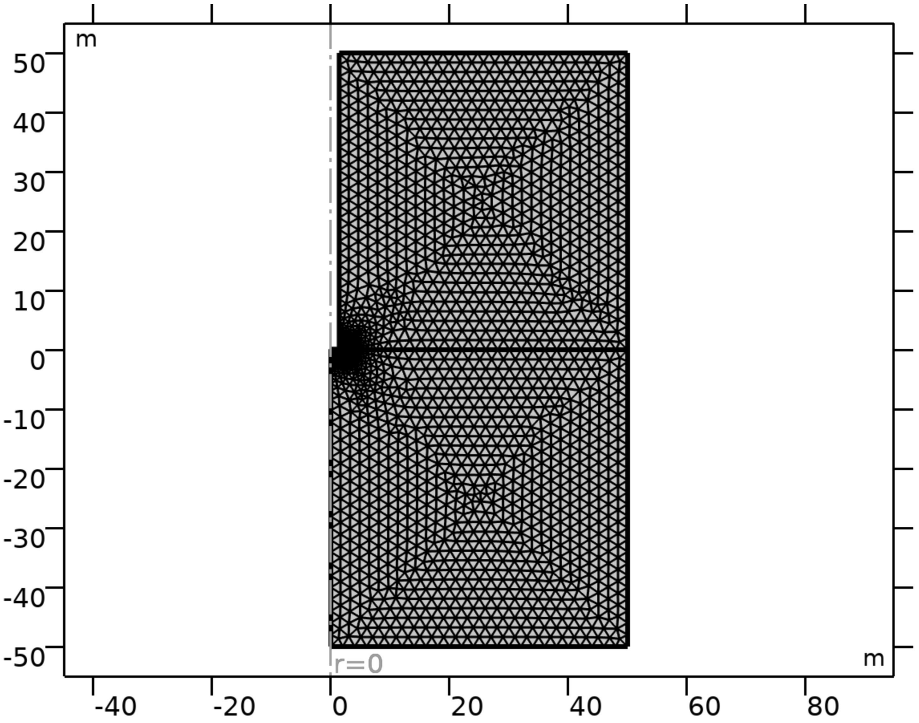

Mesh 1

3. Study 1

Computation information

Computation time	2 min 33 s

3.1. Parametric Sweep

Parameter name	Parameter value list	Parameter unit
L	range(10,-0.1,0.1)	m

Study settings

Description	Value
Sweep type	Specified combinations
Parameter name	L
Unit	m

Parameters

Parameter name	Parameter value list	Parameter unit
L (normalized tip-membrane distance)	range(10,-0.1,0.1)	m

3.2. Stationary

Study settings

Description	Value
Include geometric nonlinearity	Off

Physics and variables selection

Physics interface	Solve for	Equation form
Transport of Diluted Species (tds)	On	Automatic (Stationary)
Transport of Diluted Species 2 (tds2)	On	Automatic (Stationary)
General Form Boundary PDE (gb)	On	Automatic (Stationary)

Store in output

Interface	Output	Selection
Transport of Diluted Species (tds)	Physics controlled	
Transport of Diluted Species 2 (tds2)	Physics controlled	
General Form Boundary PDE (gb)	Physics controlled	

Mesh selection

Component	Mesh
Component 1	Mesh 1

4. Study 2

Computation information

Computation time	11 s

4.1. Time Dependent

Times	Unit
range(0,0.1,10)	s

Study settings

Description	Value
Include geometric nonlinearity	Off

Study settings

Description	Value
Output times	{0, 0.1, 0.2, 0.3, 0.4, 0.5, 0.6, 0.7, 0.8, 0.9, 1, 1.1, 1.2, 1.3, 1.4, 1.5, 1.6, 1.7, 1.8, 1.9, 2, 2.1, 2.2, 2.3, 2.4, 2.5, 2.6, 2.7, 2.8, 2.9, 3, 3.1, 3.2, 3.3, 3.4, 3.5, 3.6, 3.7, 3.8, 3.9, 4, 4.1, 4.2, 4.3, 4.4, 4.5, 4.6, 4.7, 4.8, 4.9, 5, 5.1, 5.2, 5.3, 5.4, 5.5, 5.6, 5.7, 5.8, 5.9, 6, 6.1, 6.2, 6.3, 6.4, 6.5, 6.6, 6.7, 6.8, 6.9, 7, 7.1, 7.2, 7.3, 7.4, 7.5, 7.6, 7.7, 7.8, 7.9, 8, 8.1, 8.2, 8.3, 8.4, 8.5, 8.6, 8.7, 8.8, 8.9, 9, 9.1, 9.2, 9.3, 9.4, 9.5, 9.6, 9.7, 9.8, 9.9, 10}

Physics and variables selection

Physics interface	Solve for	Equation form
Transport of Diluted Species (tds)	On	Automatic (Time dependent)
Transport of Diluted Species 2 (tds2)	On	Automatic (Time dependent)
General Form Boundary PDE (gb)	On	Automatic (Time domain)

Store in output

Interface	Output	Selection
Transport of Diluted Species (tds)	Physics controlled	
Transport of Diluted Species 2 (tds2)	Physics controlled	
General Form Boundary PDE (gb)	Physics controlled	

Mesh selection

Component	Mesh
Component 1	Mesh 1

5. Results

5.1. Data Sets

5.1.1. Study 1/Solution 1

Solution

Description	Value
Solution	Solution 1 (sol1)
Component	Component 1 (comp1)

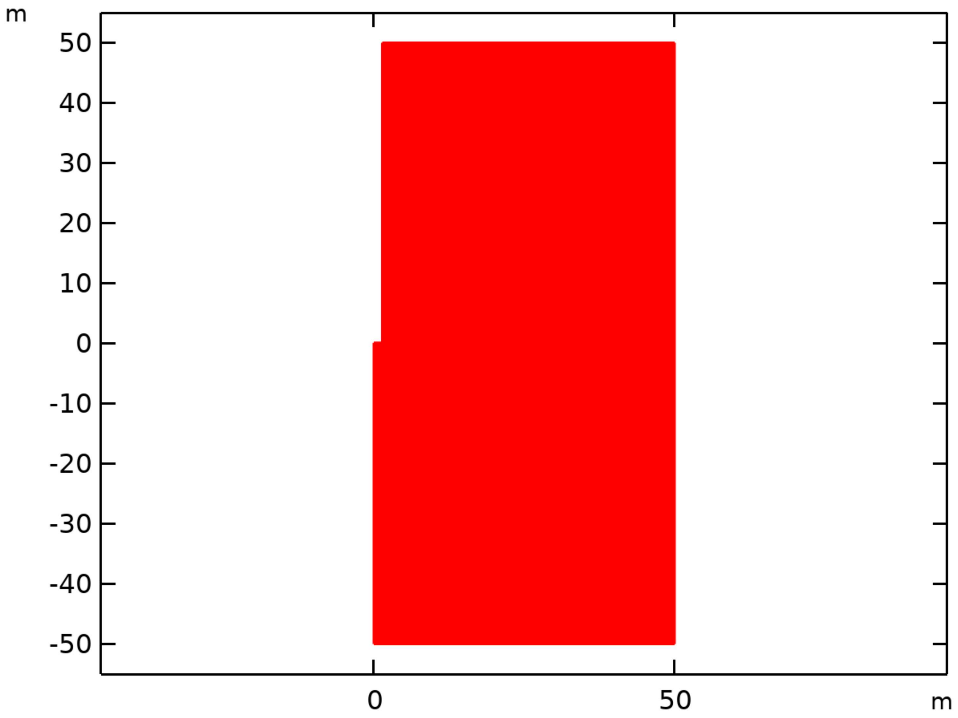

Dataset: Study 1/Solution 1

5.1.2. Revolution 2D

Data

Description	Value
Dataset	Study 1/Solution 1 (sol1)

Axis data

Description	Value
Axis entry method	Two points
Points	{{0, 0}, {0, 1}}

Revolution layers

Description	Value
Start angle	-90
Revolution angle	225

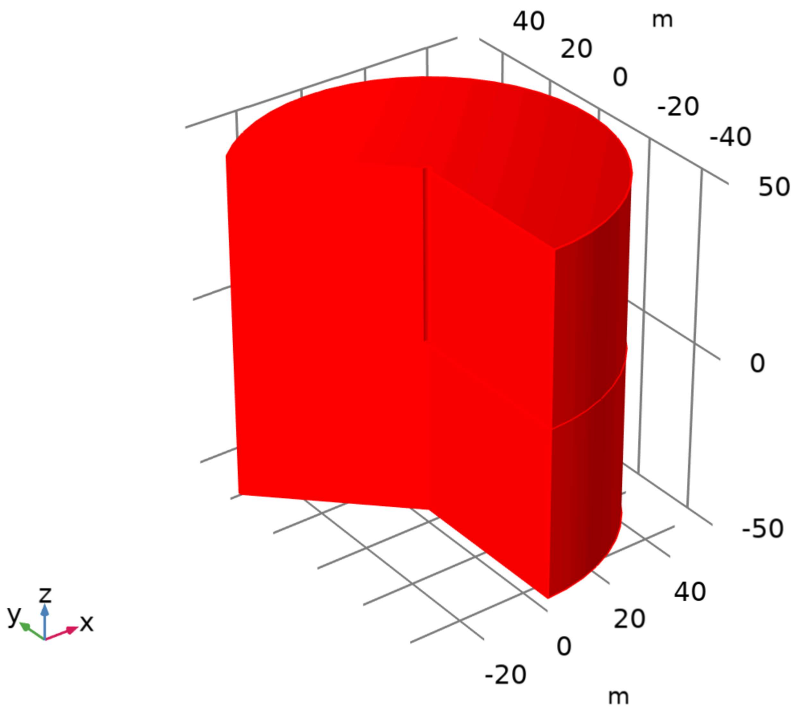

Dataset: Revolution 2D

5.1.3. Probe Solution 2

Solution

Description	Value
Solution	Solution 173 (sol173)
Component	Component 1 (comp1)

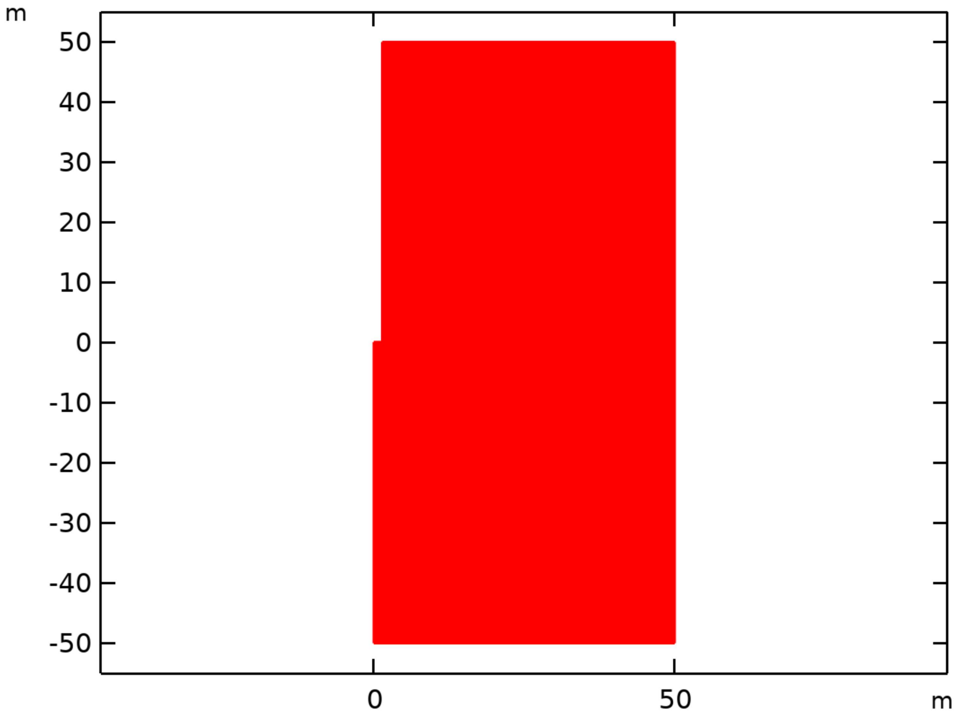

Dataset: Probe Solution 2

5.1.4. Boundary Probe 1

Selection

Geometric entity level	Boundary
Selection	Geometry geom1: Dimension 1: Boundary 5

Data

Description	Value
Dataset	Probe Solution 2 (sol173)

Settings

Description	Value
Method	Integration
Integration order	4
Integration order	On

5.1.5. Study 1/Parametric Solutions 1

Solution

Description	Value
Solution	Parametric Solutions 1 (sol2)
Component	Component 1 (comp1)

5.1.6. Revolution 2D 2

Data

Description	Value

Dataset	Study 1/Parametric Solutions 1 (sol2)

Axis data

Description	Value
Axis entry method	Two points
Points	{{0, 0}, {0, 1}}

Revolution layers

Description	Value
Start angle	-90
Revolution angle	225

5.1.7. Study 1/Parametric Solutions 2

Solution

Description	Value
Solution	Parametric Solutions 2 (sol53)
Component	Component 1 (comp1)

5.1.8. Revolution 2D 3

Data

Description	Value
Dataset	Study 1/Parametric Solutions 2 (sol53)

Axis data

Description	Value
Axis entry method	Two points
Points	{{0, 0}, {0, 1}}

Revolution layers

Description	Value
Start angle	-90
Revolution angle	225

5.1.9. Study 1/Parametric Solutions 3

Solution

Description	Value
Solution	Parametric Solutions 3 (sol72)
Component	Component 1 (comp1)

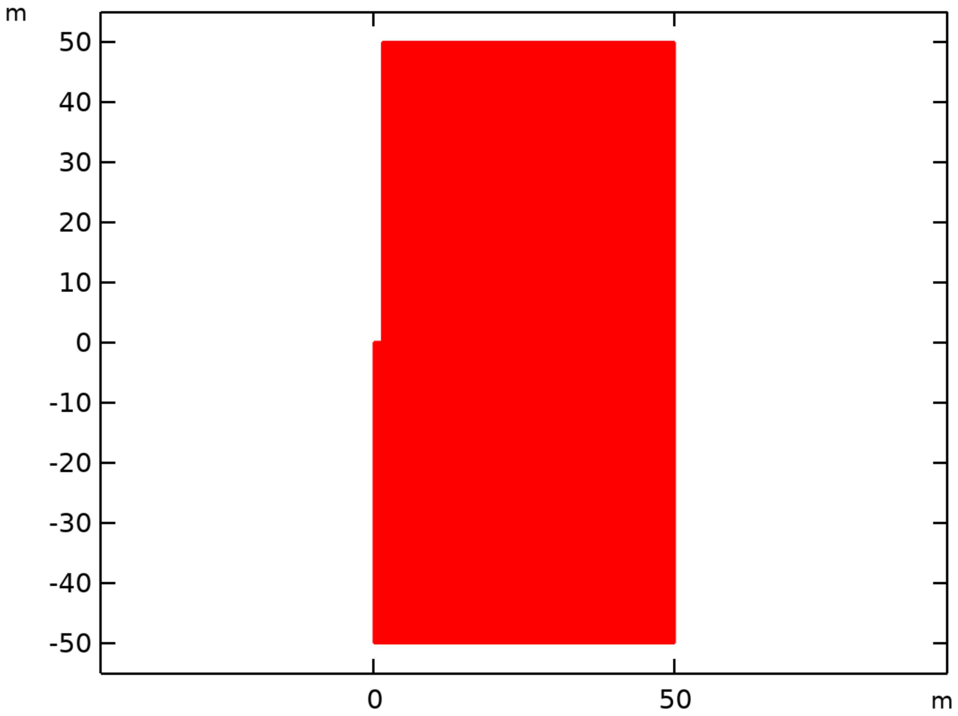

Dataset: Study 1/Parametric Solutions 3

5.1.10. Revolution 2D 4

Data

Description	Value
Dataset	Study 1/Parametric Solutions 3 (sol72)

Axis data

Description	Value
Axis entry method	Two points
Points	{{0, 0}, {0, 1}}

Revolution layers

Description	Value
Start angle	-90
Revolution angle	225

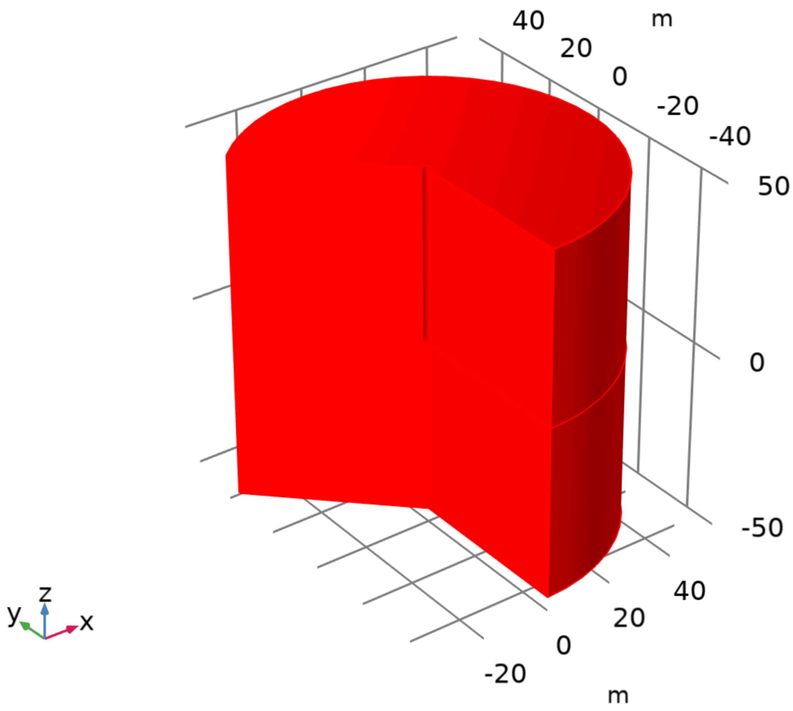

Dataset: Revolution 2D 4

5.1.11. Study 2/Solution 173

Solution

Description	Value
Solution	Solution 173 (sol173)
Component	Component 1 (comp1)

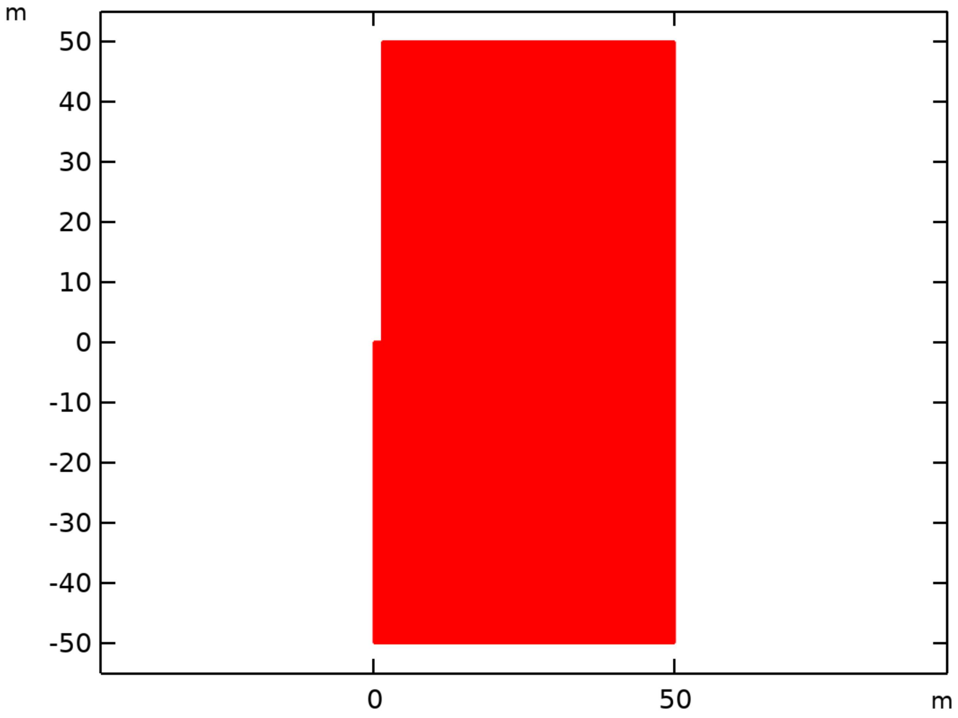

Dataset: Study 2/Solution 173

5.1.12. Revolution 2D 5

Data

Description	Value
Dataset	Study 2/Solution 173 (sol173)

Axis data

Description	Value
Axis entry method	Two points
Points	{{0, 0}, {0, 1}}

Revolution layers

Description	Value
Start angle	-90
Revolution angle	225

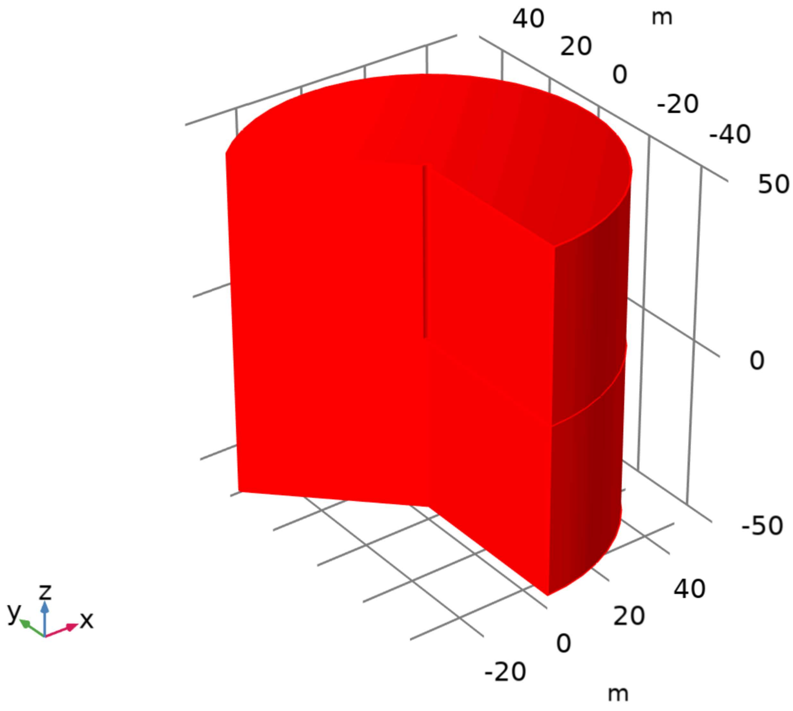

Dataset: Revolution 2D 5

5.2. Derived Values

5.2.1. Boundary Probe 1

Output

Evaluated in	Probe Table 1

Data

Description	Value
Dataset	Boundary Probe 1

Expressions

Expression	Unit	Description

intop1(tds.ndflux_c1)	mol/s	

5.3. Tables

5.3.1. Probe Table 1

Time (s)	intop1(tds.ndflux_c1) (mol/s), Boundary Probe 1
0	412.58
2.1012E-5	401.02
2.2024E-5	390.04
2.4048E-5	369.74
2.8096E-5	337.33
3.6192E-5	291.84
4.4289E-5	262.49
5.2385E-5	241.59
6.0481E-5	225.46
7.6674E-5	200.45
9.2866E-5	182.66
1.0906E-4	169.22
1.2525E-4	158.55
1.5764E-4	141.77
1.9002E-4	129.58
2.2241E-4	120.26
2.5479E-4	112.81
3.1956E-4	101.08
3.8433E-4	92.511
4.491E-4	85.941
5.1387E-4	80.679
6.4341E-4	72.382
7.7295E-4	66.318
9.0249E-4	61.664
0.001032	57.935
0.0011616	54.844
0.0014206	49.805
0.0016797	45.917
0.0019388	42.754
0.0021979	40.054
0.002716	35.416
0.0032342	31.564
0.0037524	28.256
0.0047887	22.767

0.005825	18.554
0.0068613	15.323
0.0078976	12.845
0.0089339	10.941
0.0099703	9.4748
0.011007	8.3406
0.012043	7.4594
0.013079	6.771
0.015152	5.7652
0.017224	5.1286
0.019297	4.7207
0.02137	4.4503
0.023442	4.262
0.025515	4.1234
0.02966	3.9171
0.033806	3.7764
0.042096	3.5737
0.050387	3.4419
0.058677	3.3472
0.066968	3.2747
0.075258	3.2174
0.091839	3.1297
0.10842	3.0682
0.125	3.0222
0.14158	2.9852
0.17474	2.9238
0.20791	2.8742
0.27423	2.7912
0.34056	2.7245
0.4732	2.6181
0.60585	2.5402
0.7385	2.4817
0.87115	2.4369
1.0038	2.402
1.2691	2.3502
1.5344	2.3162
1.7997	2.2931
2.065	2.2767
2.3303	2.2645
2.8609	2.2464

3.3915	2.2344
4.3915	2.2188
5.3915	2.2091
6.3915	2.2023
7.3915	2.1971
8.3915	2.1929
9.3915	2.1894
10.391	2.1864

5.3.2. Evaluation 2D

Interactive 2D values

x	y	Value
2.8929	-0.21973	0.93422
0.33462	0.091346	-0.0086338
0.34174	0.012952	-0.086873
0.14762	0.68869	0.19022
0.73637	0.36524	0.92672
0.48924	0.66689	0.82101
0.48924	0.67779	0.81734
0.54042	1.9754	0.024472
0.84368	0.1007	1.0062
0.33573	0.46712	0.95784

5.4. Plot Groups

5.4.1. Probe Plot Group 1

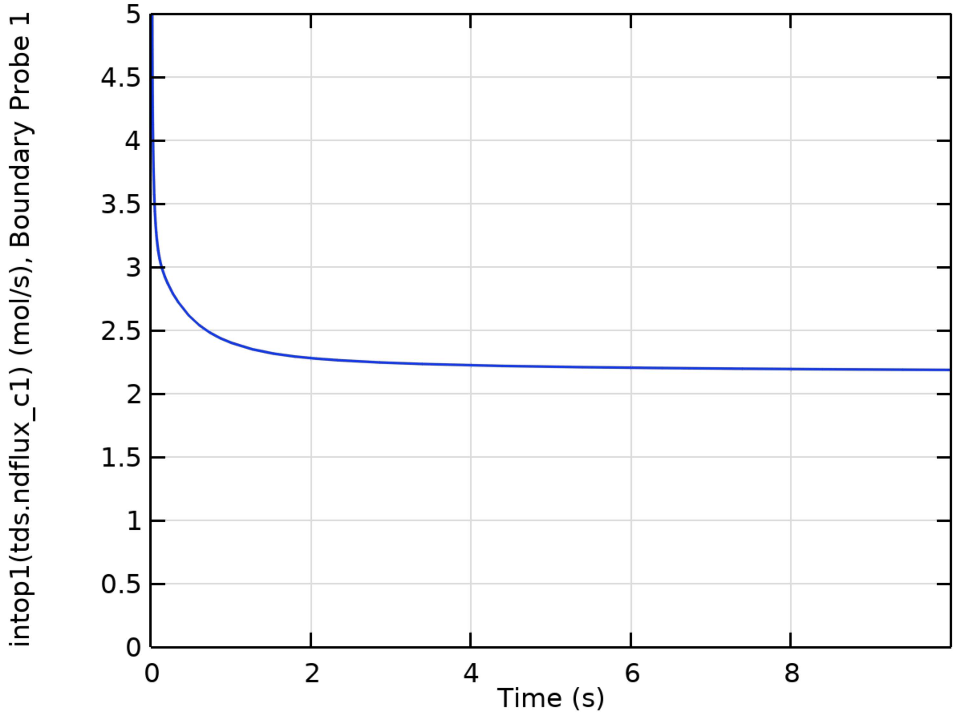

5.4.2. Concentration (tds)

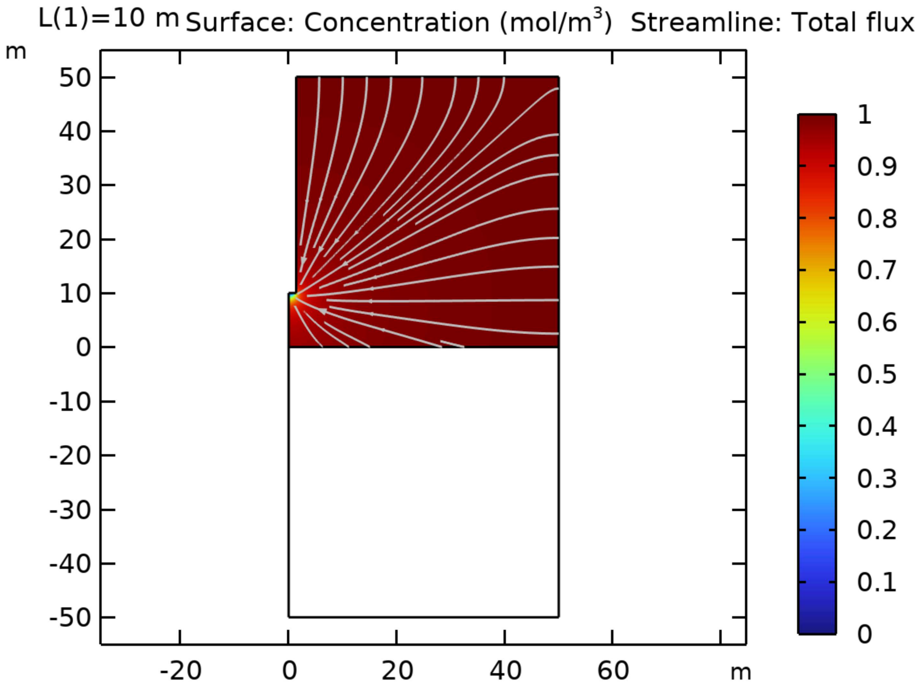

Surface: Concentration (mol/m3) Streamline: Total flux

5.4.3. Concentration, 3D (tds)

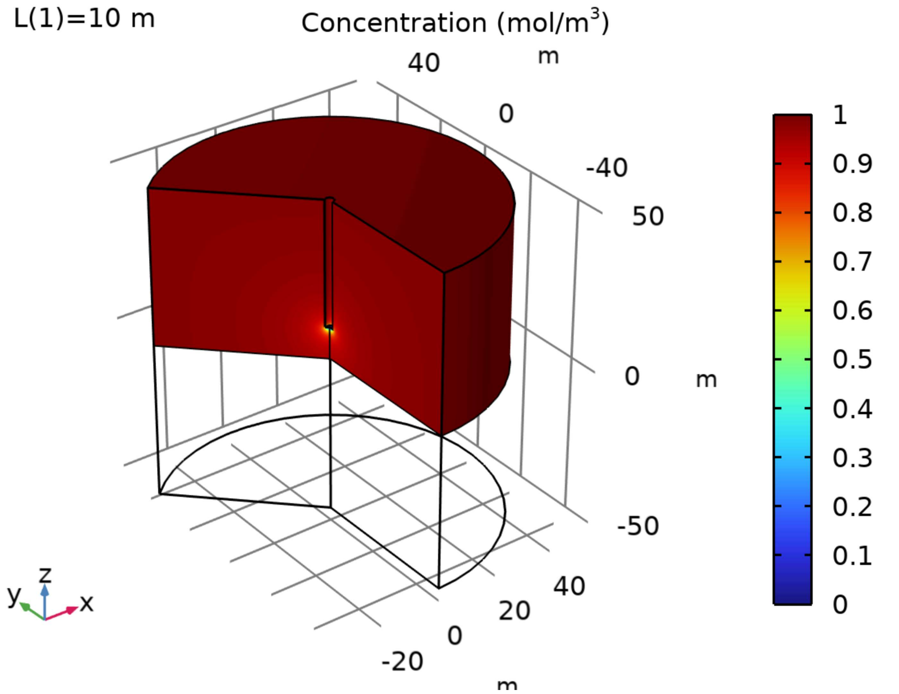

Concentration (mol/m3)

5.4.4. Concentration (tds2)

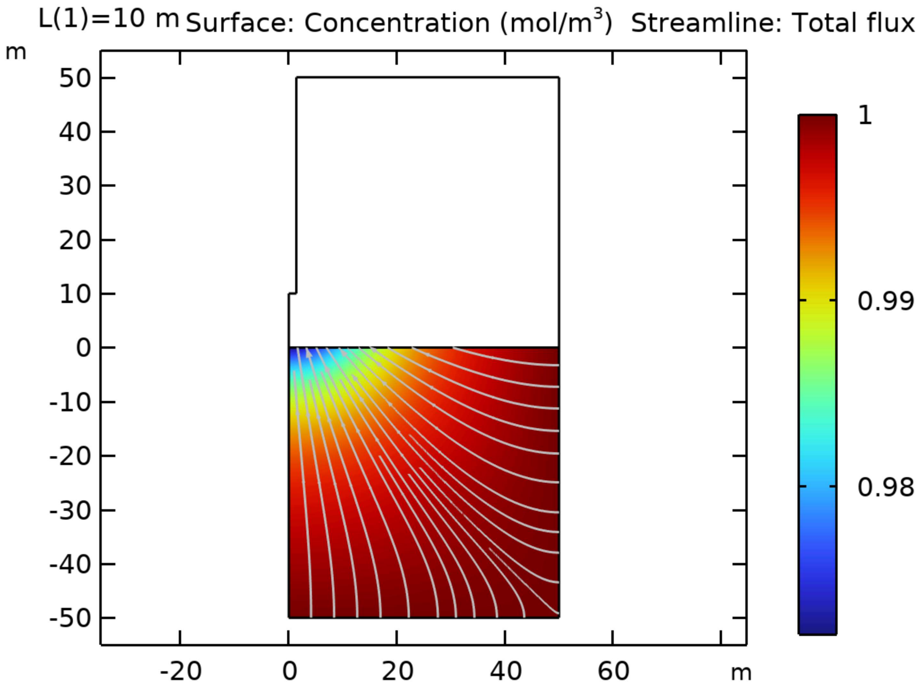

Surface: Concentration (mol/m3) Streamline: Total flux

5.4.5. Concentration, 3D (tds2)

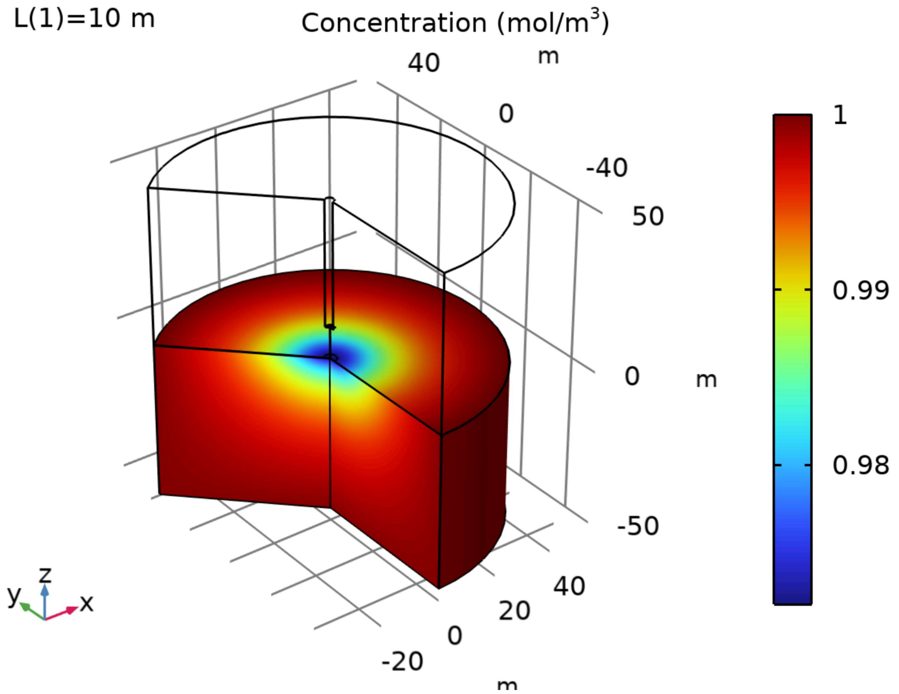

Concentration (mol/m3)

5.4.6. General Form Boundary PDE

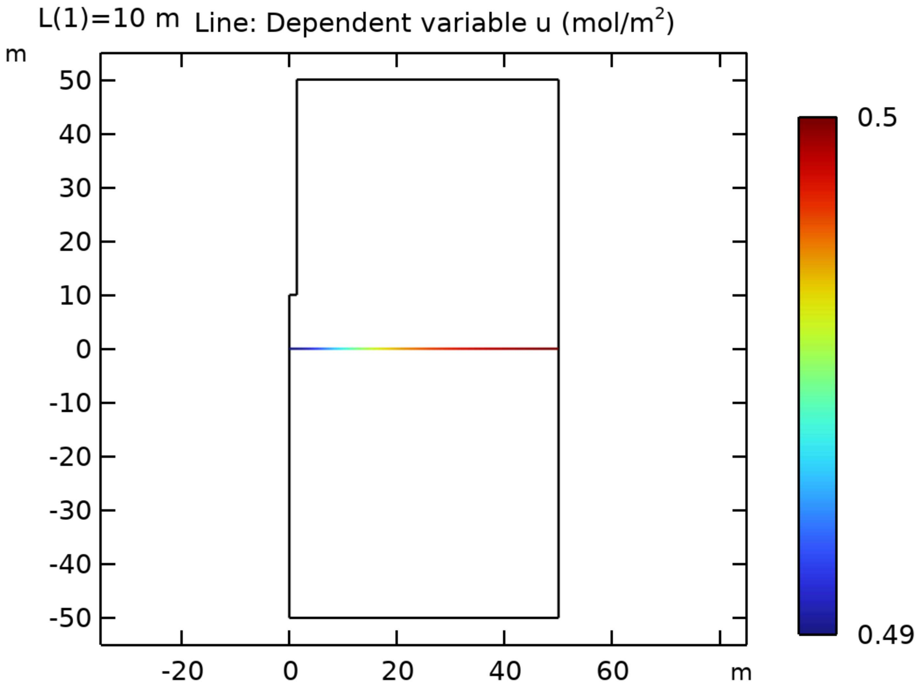

Line: Dependent variable u (mol/m2)

5.4.7. Concentration (tds) 1

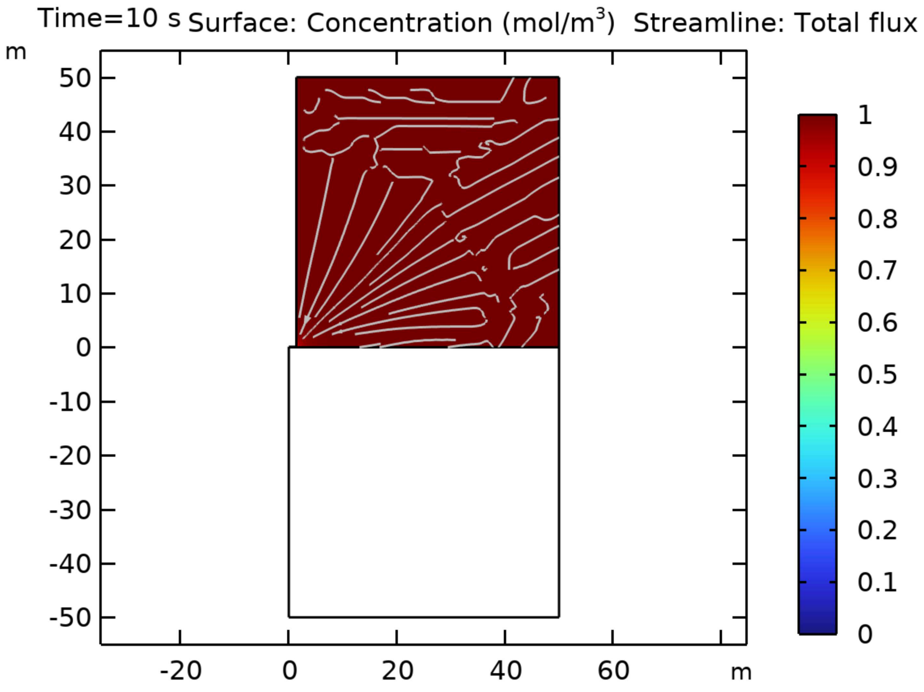

Surface: Concentration (mol/m3) Streamline: Total flux

5.4.8. Concentration, 3D (tds) 1

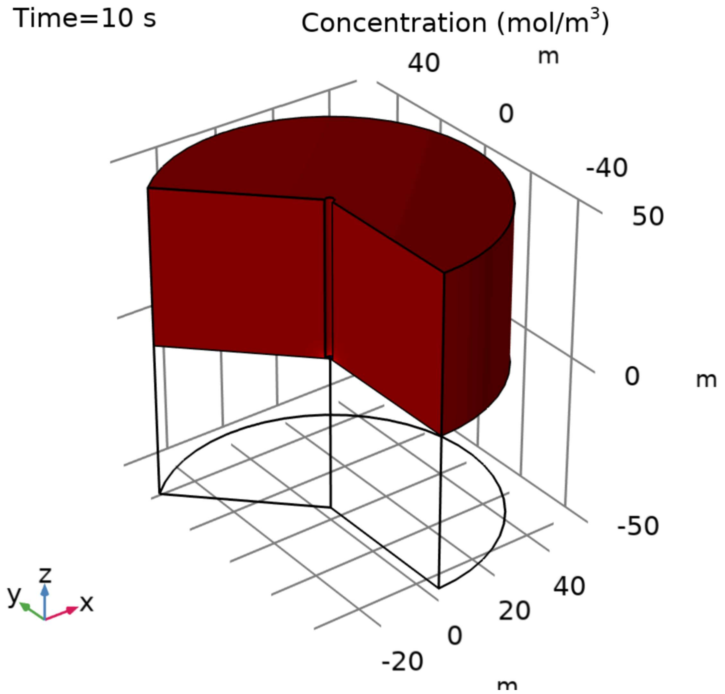

Concentration (mol/m3)

5.4.9. Concentration (tds2) 1

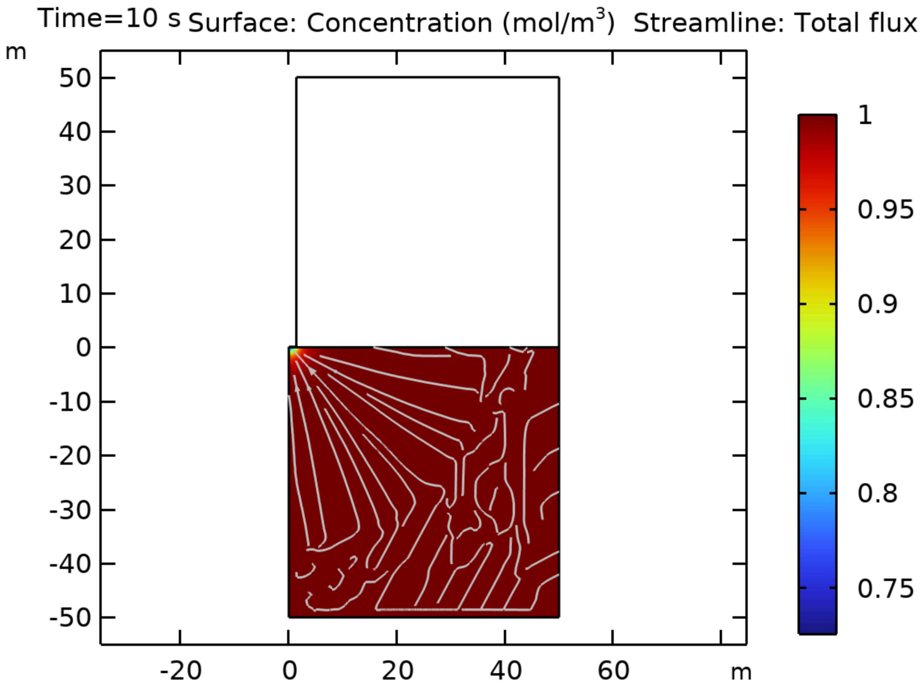

Surface: Concentration (mol/m3) Streamline: Total flux

5.4.10. Concentration, 3D (tds2) 1

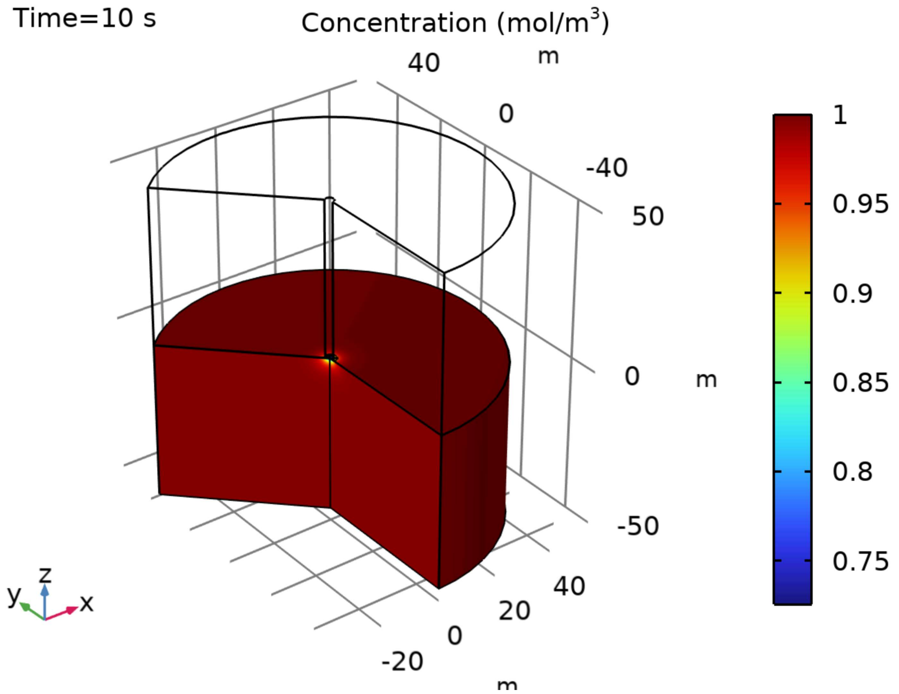

Concentration (mol/m3)

5.4.11. General Form Boundary PDE 1

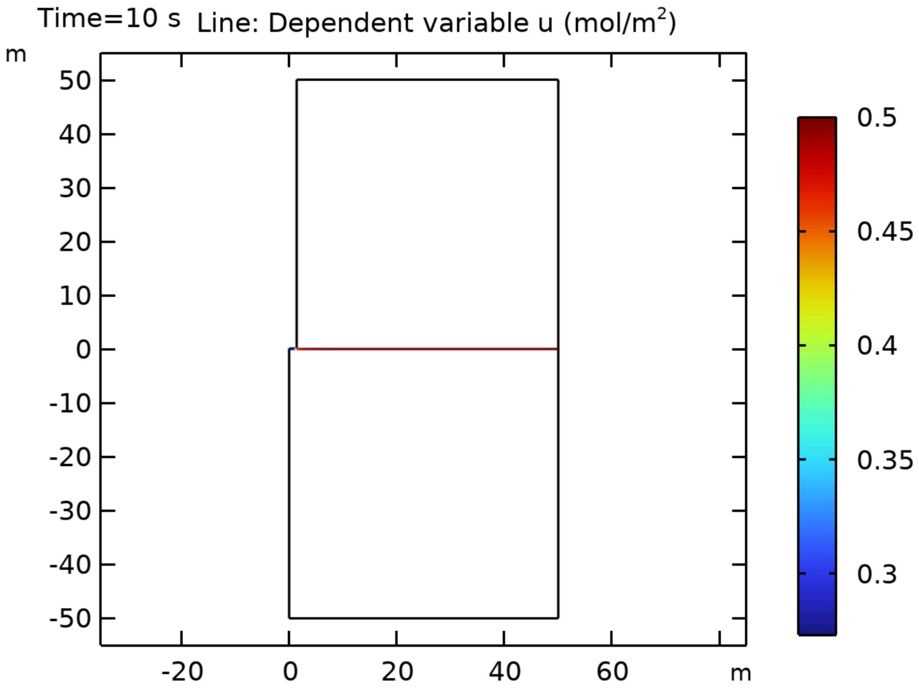

Line: Dependent variable u (mol/m2)
